# Sequential Formation of Heteroternary Cucurbit[10]uril (CB[10]) Complexes

**DOI:** 10.1002/chem.202201656

**Published:** 2022-09-14

**Authors:** Chunyang Li, Anne‐Doriane Manick, Yuxi Zhao, Fengbo Liu, Bastien Chatelet, Roselyne Rosas, Didier Siri, Didier Gigmes, Valerie Monnier, Laurence Charles, Julie Broggi, Simin Liu, Alexandre Martinez, Anthony Kermagoret, David Bardelang

**Affiliations:** ^1^ Aix Marseille Univ, CNRS Centrale Marseille, iSm2 UMR7313, AMUTech 13397 Marseille France; ^2^ School of Materials Science and Engineering Sichuan University of Science & Engineering Zigong 643000 P. R. China; ^3^ Material Corrosion and Protection Key Laboratory of Sichuan Province Sichuan University of Science & Engineering Zigong 643000 P. R. China; ^4^ Aix Marseille Univ, CNRS, ICR, AMUTech 13397 Marseille France; ^5^ School of Chemistry and Chemical Engineering Wuhan University of Science and Technology Wuhan 430081 P. R. China; ^6^ Aix Marseille Univ, CNRS, Spectropole FR 1739 Marseille France

**Keywords:** azaphosphatrane, CB[10], cucurbituril, host:guest, ternary complexes

## Abstract

The globular and monocationic guest molecule trimethyl‐azaphosphatrane (**AZAP**, a protonated Verkade superbase) was shown to form a host:guest 1 : 1 complex with the cucurbit[10]uril (CB[10]) macrocycle in water. Molecular dynamics calculations showed that CB[10] adopts an 8‐shape with **AZAP** occupying the majority of the internal space, CB[10] contracting around **AZAP** and leaving a significant part of the cavity unoccupied. This residual space was used to co‐include planar and monocationic co‐guest (**CG**) molecules, affording heteroternary CB[10]⋅**AZAP**⋅**CG** complexes potentially opening new perspectives in supramolecular chemistry.

## Graphical abstract

**An azaphosphatrane (AZAP**) forms a host:guest 1 : 1 complex with the cucurbit[10]uril (CB[10]) macrocycle in water and forces CB[10] to adopt an 8‐shape with **AZAP** occupying the majority of the cavity. The CB[10] contraction leads to a residual space which favors the co‐inclusion of planar and monocationic co‐guest (**CG**) molecules, affording heteroternary CB[10]⋅**AZAP**⋅**CG** complexes.

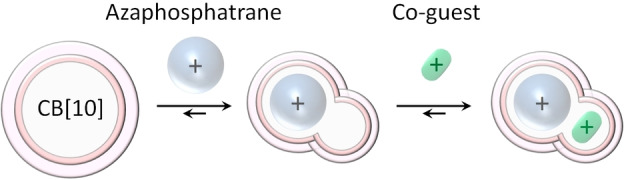

## Introduction

**Figure 1:**
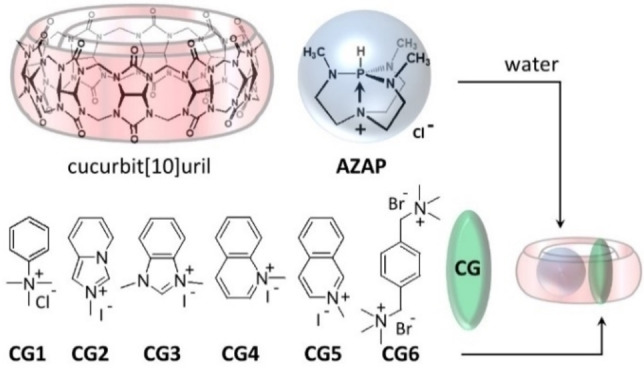
(a) Molecular structures of CB[10], **AZAP**, and co‐guest (**CG1** to **CG6**) molecules used in this work.

Ternary assemblies of proteins are known to play crucial roles in cells. By assembling in particular oligomers of controlled size and shape, new functions emerged surpassing what could be done based on individual components. For example, ternary complexes of proteins are involved in voltage‐gated potassium channels that are pivotal for channels functions in the brain and in the heart.^[1]^ Heterotrimeric G proteins can function as transducers in signaling pathways,^[2]^ or bind the adenosine A1 receptor as illustrated in the search for non‐opioid analgesic agents.^[3]^ However, artificial heteroternary complexes are still limited to comparatively small molecules and these include for example metal‐ligand cages accommodating two types of guests,^[4]^ molecular Russian dolls,^[5]^ or organic cages with different guest molecules.^[6]^ In this context, the cucurbit[8]uril (CB[8]) macrocycle^[7]^ was shown in 2001 to be able to form heteroternary complexes^[8]^ with a pair of guest molecules combining electron donor and electron acceptor features.^[9]^ This discovery has opened a new domain by enabling to “click” by CB[8], two types of compounds (i. e. peptides,^[10]^ proteins,^[11]^ polymers,^[12]^ dendrimers)^[13]^ each carrying a suitable group for inclusion in CB[8]. Recent developments include molecular switches,^[14]^ host‐enhanced polar‐π interactions (social self‐sorting),^[15]^ dynamic oligomers,^[16]^ bifunctional photoredox catalysts,^[17]^ curcumin delivery in cancer cells,^[18]^ advanced herbicides^[19]^ or dynamic interfacial adhesion.^[20]^ Compared to the more traditional “covalent click” chemistry, this method is endowed with inherent dynamics while permitting to get sufficiently robust links. After the discovery of cucurbit[10]uril,^[21]^ and of its isolation,^[22]^ several studies have demonstrated its unique potential in supramolecular chemistry^[23]^ owing to its large open cavity. During our investigations about new guest molecules of interest for CB[10],^[24]^ we found that trimethyl‐azaphosphatrane (**AZAP**, a protonated Verkade superbase,^[25]^ Figure 1) could form 1 : 1 complexes with CB[10], the host cavity being left with enough space for other compounds.

Azaphosphatranes are the conjugated acids, of the well‐known Verkade superbases (pro‐azaphosphatranes).^[25]^ They are the subject of growing interest and were found to act as efficient organocatalysts in various reactions such as ring‐opening polymerization (ROP), Strecker reactions, or for CO_2_ conversion into cyclic carbonates.^[26]^ The properties of self‐ assembled or covalent cages built from azaphosphatrane units^[27]^ have been also studied and, for example, self‐assembled cages involving azaphosphatrane motifs as sub‐components can recognize and extract anions from water with remarkable selectivity.^[28]^ However only one example of host‐guest complex where an azaphosphatrane plays the role of guest has been described leading to modification of acido/basic properties of the encaged guest.^[29]^ Thus, the supramolecular encapsulation of azaphosphatranes and the properties of the resulting host‐guest complexes have been scarcely studied and could lead to new supramolecular objects with original properties. On the other hand, few heteroternary complexes based on CB[10] have been reported, including a calixarene,^[22]^ a Russian doll,^[30]^ and tetracationic porphyrins.^[31]^ Given the impact of CB[8] on supramolecular coupling by forming heteroternary complexes, we focused on (i) finding plausible reasons to explain the present results, and (ii) deciphering the scope of co‐guests (**CG**) suitable to form CB[10]⋅**AZAP**⋅**CG** heteroternary complexes.

## Results and Discussion

**Figure 2:**
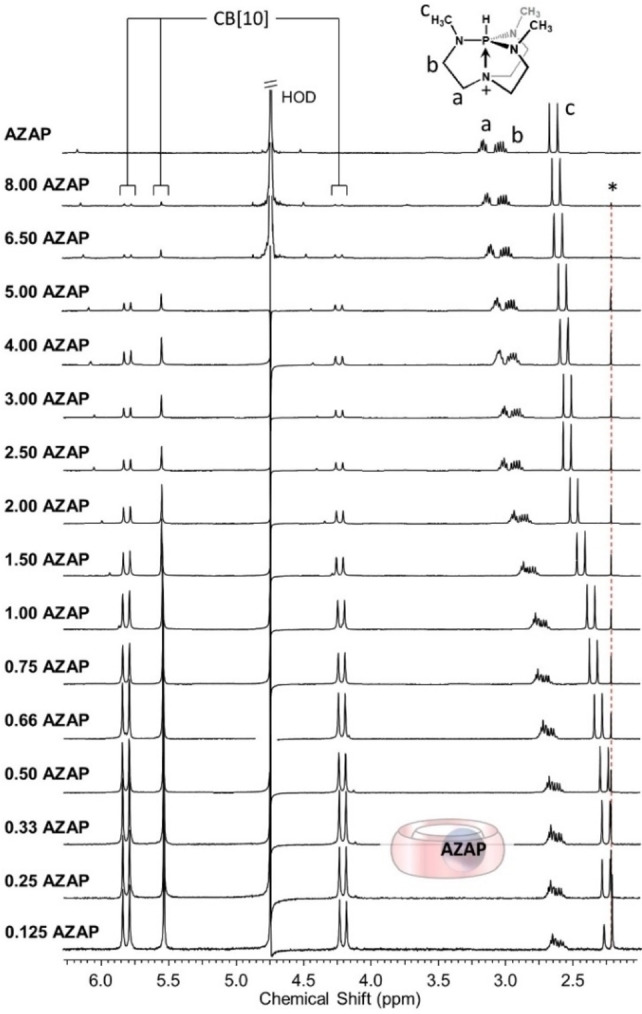
^1^H NMR titration (300 MHz, 300 K, D_2_O) of **AZAP** keeping constant the quantity of CB[10] in the tubes (around 0.41 mg of CB[10] in 500 μL of D_2_O see experimental section, guest:host ratios on the left). *signal of acetone.

While exploring new guests for CB[10], we found that **AZAP** could solubilize CB[10] (otherwise scarcely soluble alone in water), and that a titration gradually changing the guest:host ratio (see Supporting Information for details) showed rapid exchange on the NMR timescale in D_2_O (Figure 2).

**Figure 3:**
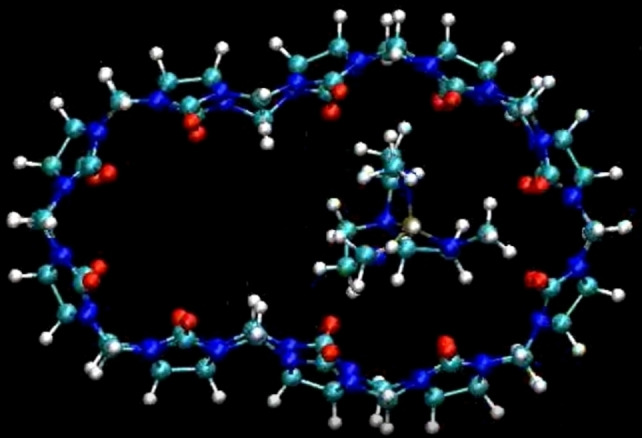
Representative snapshot of the CB[10]⋅**AZAP** complex from the corresponding molecular dynamics in water (solvent removed for clarity).

In this series of experiments, the chemical shift of guest methyl protons shifted upfield by 0.40 ppm (Hc) while those corresponding to guest methylene protons also shifted upfield but by 0.42 ppm (Hb) and 0.50 ppm (Ha, Figure 2). ^31^P NMR spectra of CB[10]⋅**AZAP** were unusable. Unexpectedly, an excess of host, even apparently insoluble, was required to get the maximum upfield shifts of guest proton signals. In principle, any undissolved CB[10] should not impact the complexation equilibrium between CB[10] and **AZAP**. However, this is what observed, repeatedly, over time, and host:guest ratios. To try understanding this observation, we have recorded ^1^H NMR spectra of CB[10] (Figure S1) in D_2_O (fixed volume) at 500 MHz using a small but precise fraction of acetone as internal standard and increased the total quantity of CB[10] (0.2 to 1.0 mg, Figure S2). As expected, the integrated ratio of signals of CB[10] compared to those of acetone remained about constant enabling to propose a concentration of CB[10] of 5.4 μM in D_2_O (Figure S3). Despite our efforts, this effect remains unexplained. We suppose a “non‐classical” dissolution/complexation of CB[10] mediated by the presence of CB[10] aggregates. Initial tests aimed at removing the excess of insoluble host by filtration resulted in different spectra presumably due to equilibria displacements. The effect of centrifugation was also checked but identical spectra were obtained without centrifugation. To go further, we performed our experiments using a “standardized” procedure involving an identical concentration of CB[10] for all the titrations of the paper (0.83 mg/mL in D_2_O). At excess host, integrals suggested formation of a 1 : 1 CB[10]⋅**AZAP** complex (Figure S4). The binding constant corresponding to this equilibrium could not be determined due to the very low solubility of CB[10] in water. Molecular dynamics calculations considering CB[10] with one included **AZAP** showed that the 1 : 1 complex is stable for at least 100 ns in water (Figure 3).

The **AZAP** guest molecule was found to be relatively weakly mobile in the CB[10] cavity that seemed largely accommodating its shape to encircle the guest (mean distance between the barycentre of the host and that of the guest oscillating between ∼2.0 and 3.5 Å, Figure S5 and video S1). The observed, asymmetric 8‐shape of CB[10] was quite stable over all the duration of the dynamics (100 ns), even if **AZAP** could travel in the host cavity (Figure S6). **AZAP** seemed entailing CB[10] to contract around it, leaving a small space amenable for further binding. By doing so, **AZAP** could have preorganized the cavity for subsequent co‐guest accommodation in an allosteric or cooperative manner. However, this is not reflected in ^1^H NMR spectra that are in line with a preserved high symmetry and suggesting that if the host is contracted, this must be averaged with respect to the NMR timescale.

The free space left available was next used to investigate co‐guest binding with a series of planar cationic compounds.

**Figure 4:**
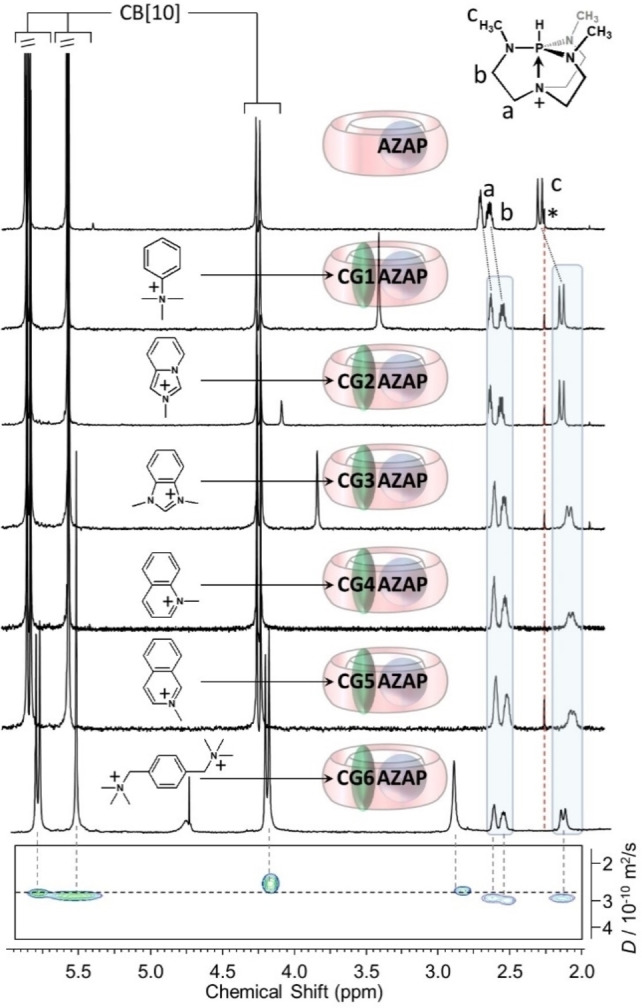
^1^H NMR spectra (600 MHz, 300 K, D_2_O) of the CB[10]⋅**AZAP** 1 : 1 complex (around 0.41 mg of CB[10] in 500 μL, see experimental section) and of heteroternary complexes CB[10]⋅**AZAP**⋅**CG1** to CB[10]⋅**AZAP**⋅**CG6** (*: signal of acetone, vertical dashed line at 2.22 ppm, CB[10] with 0.3 equiv. of **AZAP** and 0.3 equiv. of **CGx** to ensure having enough excess host). Bottom, DOSY spectrum corresponding to the CB[10]⋅**AZAP**⋅**CG6** complex.

The addition of planar and monocationic guest compounds **CG1** to **CG5** to solutions of the CB[10]⋅**AZAP** complex (containing a CB[10]/**AZAP** ratio of 1/0.3, to ensure having a fully complexed **AZAP**, see experimental part) showed extra‐upfield shifts of **AZAP** resonances (ΔδHa=−0.01 to −0.10 ppm, ΔδHb=−0.01 to −0.12 ppm, ΔδHc=−0.08 to −0.23 ppm, Figure 4) supporting that no decomplexation of **AZAP** occurred upon addition of the second guests. For dicationic guests, dimethyl‐viologen or hexamethyl‐*N*,*N*‐bisethylene‐1,4,5,8‐naphthalenediimide diammonium, two guests known to include in CB[10]^[32]^ had no impact on the ^1^H NMR spectrum of the CB[10]⋅**AZAP** complex, while hexamethyl‐*p*‐xylylenediammonium (**CG6**) did (Figure 4 bottom).

DOSY NMR of the CB[10]⋅**AZAP** complex (Figure S7) showed all host and guest signals aligned with a diffusion coefficient *D*≈2.87×10^−10^ m^2^.s^−1^ corresponding to ∼15 Å of diameter (spherical approximation) so in line with the size of CB[10]. The DOSY spectrum of CB[10]⋅**AZAP**⋅**CG6** (Figure 4, bottom) was in line with (i) double‐guest inclusion (no free guest) and (ii) a size of the CB[10]⋅**AZAP**⋅**CG6** complex close to that of CB[10] (*D*≈2.82×10^−10^ m^2^.s^−1^, inclusion complexation). DOSY spectra of other ternary complexes CB[10]⋅**AZAP**⋅**CGx** (x=1–5, Figures S8 to S12) showed signals deviating from ideal alignment due to rapid exchange of **CGx** with CB[10]⋅**AZAP**.^[33]^ Another evidence toward heteroternary complexation is the significant extra‐upfield shift of **AZAP** signals (Figure 4), in line with favored social self‐sorting (CB[10]⋅**AZAP**⋅**CGx**) compared to narcissistic self‐sorting (CB[10]⋅**AZAP** and CB[10]⋅**CGx**_2_). Controls titrating separately **CG2** and **CG6** with CB[10] (Figures S13 and S14 respectively) to explore the alternative sequence of guest binding (CB[10]⋅**CGx**+**AZAP**⇆CB[10]⋅**CGx**⋅**AZAP**) showed that 1 : 1 CB[10]⋅**CGx** complexes could form but were less favored than corresponding heteroternary complexes and we could not discard the presence of CB[10]⋅(**CGx**)_2_ complexes contrary to **AZAP** which formed exclusively CB[10]⋅**AZAP** 1 : 1 complexes.

Careful inspections showed that **AZAP** impacted **CGx** proton resonances of included **CGx** with downfield shifts for methyl groups (+0.06 or +0.09 ppm for **CG1**, **CG2** and **CG3**) suggesting that **AZAP** may help placing **CGx** methyl groups near the host carbonyl rims, while aromatic signals are weakly affected (except for **CG3**, Δδ=+0.07 and +0.11 ppm). Finally, while weak signal‐to‐noise ratios prevented the quantification of complexation induced chemical shift changes for **CG4**, one signal of **CG5** was featured by a +0.18 ppm downfield shift as a result of the simultaneous presence of **AZAP** in CB[10], compared to signals of CB[10]⋅**CG5**. Finally, compared to free **CG6**, the signal corresponding to the aromatic group of the **CG6** co‐guest in the CB[10]⋅**AZAP**⋅**CG6** complex shifted upfield by 0.42 ppm while the one corresponding to methyl groups shifted also upfield but by 0.17 ppm. The search for NOE cross‐correlations between included **AZAP** and **CGx** was unsuccessful despite the record of 2D NOESY spectra at different mixing times.

The addition of **CG2** was also tested to see whether **CG2** could promote **AZAP** capture in conditions where **AZAP** is only partially complexed in CB[10]. **CG2** was added in a solution containing CB[10] and **AZAP** in a 1 : 1 ratio (corresponding to ∼75 % of complexed **AZAP**, Figure S15) and the ^1^H NMR spectra showed an upfield shift of **AZAP** signals with 0.5 and 1 equiv. of **CG2** (Figure S15) confirming the suspected cooperativity. However, when more than 2 equiv. of **CG2** were added, both sets of NMR signals corresponding to **AZAP** and **CG2** shifted downfield as a consequence of a competition between formation of CB[10]⋅**AZAP**⋅**CG2** and CB[10]⋅(**CG2**)_n_ (n=1 or 2).

**Figure 5:**
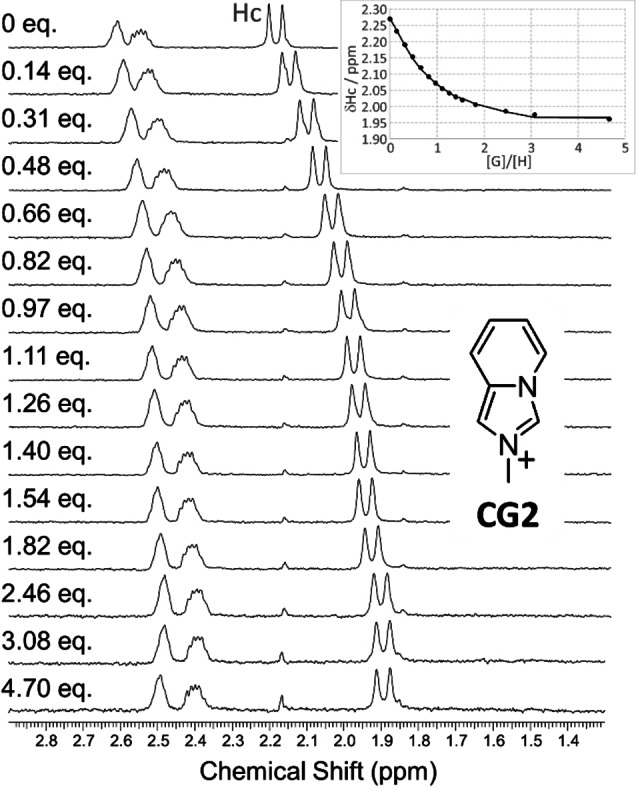
^1^H NMR titration of the CB[10]⋅**AZAP** 1 : 1 complex (around 0.41 mg of CB[10] in 500 μL of D_2_O, see experimental section) with **CG2** (500 MHz, 300 K).

Next, considering the CB[10]⋅**AZAP** complex as a “soluble modified host” (when a CB[10]:**AZAP** ratio of 1:0.3 is used), we first checked the stoichiometry of the CB[10]⋅**AZAP**⋅**CG2** complex by a Job Plot (Figure S16) confirming formation of a “1 : 1 complex” between CB[10]⋅**AZAP** and **CG2**. Then we performed ^1^H NMR titrations considering increasing amounts of co‐guest molecules (Figures S17‐S22, Table 1, for a representative example see Figure 5). Fitting experimental points considering complexation induced **AZAP** chemical shift changes according to a 1 : 1 binding model afforded binding constants *K*_a_ associated to the following equilibria: CB[10]⋅**AZAP**+**CGx**↔CB[10]⋅**AZAP**⋅**CGx** (Figure 5 inset and Table 1).

**Table 1:** Binding constants for co‐guests **CG1** to **CG5** toward the CB[10]⋅**AZAP** complex, maximum observed Δδ and mean distances from MD simulations.

CGx	*K*_a_ [M^−1^]^[a]^	Δδ_max_ [ppm]^[b]^	d AZAP‐CGx	d Host‐AZAP	d Host‐CGx
**CG1**	6800	0.23	7.1±0.3 Å	2.9±0.2 Å	4.5±0.3 Å
**CG2**	25100	0.30	7.0±0.3 Å	3.0±0.2 Å	4.1±0.3 Å
**CG3**	25000	0.43	6.5±0.6 Å	3.0±0.3 Å	3.8±0.5 Å
**CG4**	1500	0.45	6.5±0.5 Å	2.9±0.2 Å	3.8±0.5 Å
**CG5**	13600	0.49	6.2±0.6 Å	2.7±0.4 Å	3.7±0.4

[a] determined using the BINDFIT program;^[34]^ error: ±11 %. [b] experimental values from signals of protons Hc, see Figure 2.

Among them and assuming co‐guests sit axially against **AZAP** inside CB[10], the least sterically demanding co‐guests *laterally* **CG2**, **CG3** and **CG5** bind with a ∼10^4^ M^−1^ affinity, the difference between **CG4** and **CG5** being especially striking (a factor of nearly 10 times) explained solely by the position of the methyl group on the co‐guest (1‐ versus 2‐quinoline). Another control mixing separately prepared solutions of CB[10]⋅**AZAP** and CB[10]⋅**CG3** afforded a new ^1^H NMR spectrum (Figure S23) different from those of the initial complexes and in line with CB[10]⋅**AZAP**⋅**CG3**. Finally, a large excess of **CG2** did not result in **AZAP** expulsion (heteroternary complex dislocation). Attempts to grow single crystals of the complexes resulted in small single crystals and those large enough for analyses by X‐ray diffraction enabled to observe a unit cell with the host only, presumably due to high guests disordering in the host cavity. Even if these structures should be considered with extreme caution due to the high levels of disordering, the host appear flattened (roughly resembling the shape shown on Figure 3 though without methylene inversion) when crystallized from a solution of CB[10] and **AZAP**, but almost circular when **CG2** was added before solvent evaporation (Figure S24).

**Figure 6:**
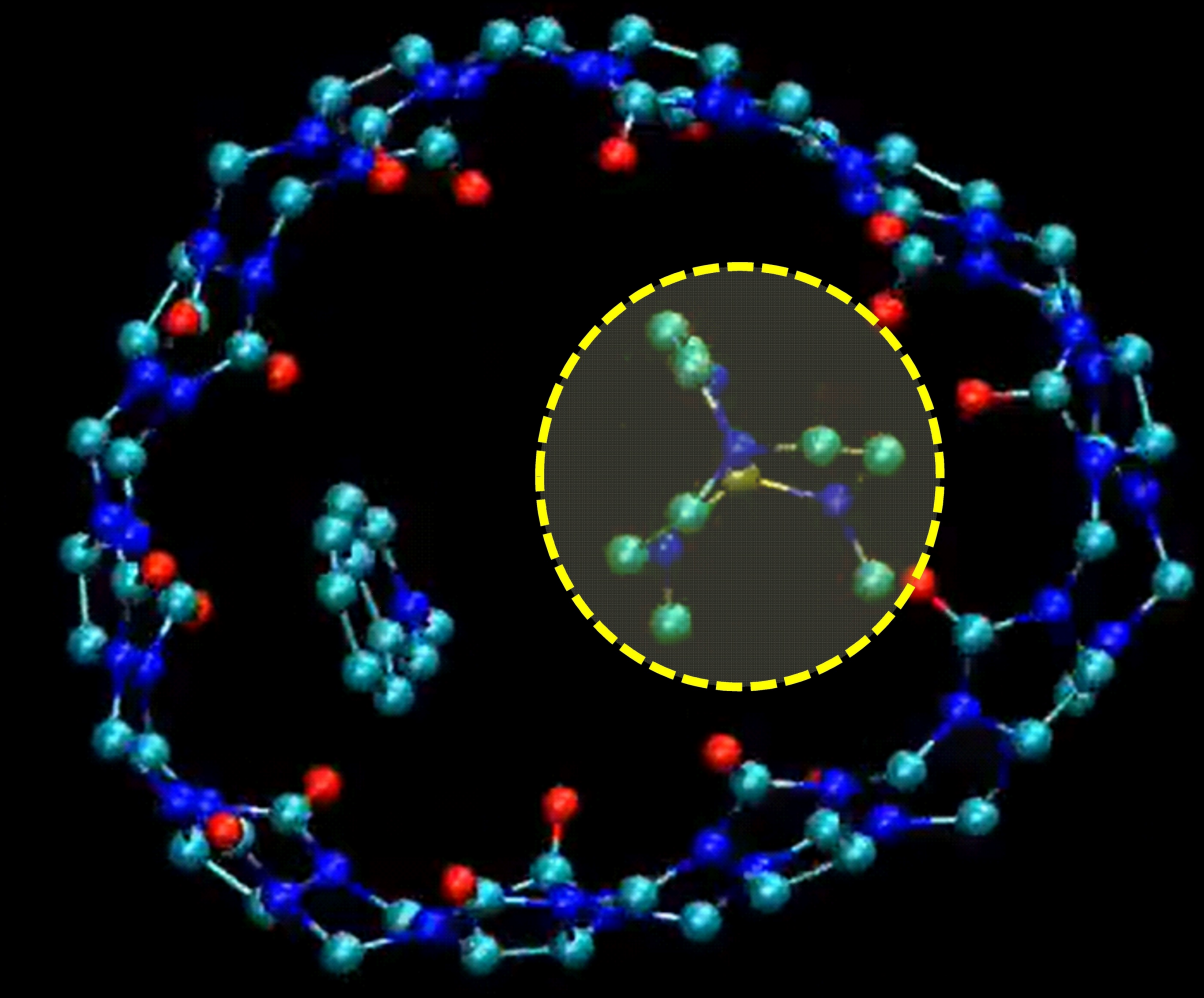
Snapshot of the CB[10]⋅**AZAP**⋅**CG5** complex from the corresponding molecular dynamics trajectory in water (hydrogen atoms removed for clarity, see text and video S2; the yellow disk delineated by a yellow dashed line denotes the **AZAP** location, the structure of CB[10] is found to be very flexible with these two included guests, see Figure S25).

We thus decided to investigate the heteroternary complexes by molecular dynamics in water. Heteroternary complexes with **CG1** to **CG5** were found to be quite stable over 100 ns in water (snapshots for the CB[10]⋅**AZAP**⋅**CG5** complex in Figure 6 and Figure S25). With co‐guest molecules, **AZAP** was found to be largely mobile in CB[10] but co‐guests could either stay inside the complex or be expulsed before being included again (this was observed for **CG3** and **CG5**). Nevertheless, in the latter case, heteroternary complexes were stable for the last 50 ns of MD trajectories. Calculations of distances between the barycentres of CB[10], **AZAP**, and **CGx** showed that the two different guests considered remained facing each other's inside CB[10] (see Supporting Information and Table 1), **AZAP** being systematically slightly offset (∼3 Å) to the center of CB[10], as **CGx** (∼4 Å). Consistently, distances between **AZAP** and **CGx** remained in the 6.3–7.3 Å window, in line with stable co‐inclusion for the 2^nd^ half of the MD trajectories (Figures S26 to S30). Attempts to quantify CB[10] deformation by measuring the 5 distances between opposite carbon atoms of the equatorial plane for CB[10]⋅**AZAP**⋅**CG1** to CB[10]⋅**AZAP**⋅**CG5** (Figure S31) showed large macrocycle contraction and expansion cycles seemingly not impacting its faculty to accommodate two of such dissimilar guest compounds. The dynamical behavior was similar for the five considered heteroternary complexes, that for **CG5** summarizing well the trends (video S2). In this case, **AZAP** was found to be highly mobile, translating and rotating in all directions in the CB[10] cavity. Simultaneously, **CG5** was found to adjust its position with respect to **AZAP**, with its main axis almost always staying parallel to the *C*_10_ axis of the host, **CG5** being able to rotate around this axis.

As a consequence, CB[10] was observed to continuously adjust its shape toward the two guests, the cause‐consequence relationship remaining difficult to firmly establish. Even if dynamic, the averaged rounded shape of CB[10] is in agreement with the shape of the host determined from the preliminary X‐ray structure. These results of host and guests dynamics in heteroternary complexes are also in line with NMR results since signals of both host and guests remain sharp and “simple” (no symmetry break), suggesting unhindered rotation. Finally, both kinds of guests experiencing a more hydrophobic environment in CB[10] should have the signals of their protons shifted upfield which is indeed observed for signals of **AZAP** protons (Figures 2, 4, 5) and **CGx** protons (i. e. Figure S15). If we consider the inclusion of **AZAP** followed by **CGx**, the former seems pre‐organizing the cavity for subsequent binding in a manner reminding an allosteric binding. However, because of the ambiguity about CB[10]⋅**CGx** complexes stoichiometry (singly or doubly accommodated guests in some cases), weak binding requiring excess guest, and fast exchange with free **CGx**, we could not investigate the equally important alternative pathway of guest inclusion (CB[10] binding **CGx** followed by **AZAP**). The question of a mechanism more relevant to induced fit (IF) or conformational selection (CS) remains open.^[35]^ In either case, the space for co‐guest inclusion (induced by **AZAP**) resembles a half‐cavity of nor‐seco‐CB[10],^[36]^ and contrasts with what reported for others CB[10] heteroternary complexes for which π‐stacking,^[31]^ or strong guest‐guest interactions involving electron donor/acceptor features^[30]^ directed the assembly. The flexibility of CB[10] could thus be an important factor in the stabilization of the reported heteroternary complexes. Globally, the observed social, instead of narcissistic self‐sorting could be explained by the preferred 1 : 1 host:guest ratio at excess host with **AZAP** resulting in formation of a CB[10]⋅**AZAP** inclusion complex featured by residual space left in the host cavity. We surmise that mixed guest pairs are favored owing to better fillings of the cavity space compared to a mixture of CB[10]⋅**AZAP** with residual internal space together with homoternary complexes featuring identical guest pairs (i. e. CB[10]⋅**CGx**⋅**CGx**) not able to fill, as well, the host cavity.

## Conclusions

In conclusion, we found that the azaphosphatrane **AZAP** could behave as a new monocationic guest for CB[10]. Beyond CB[10] solubilization and formation of a 1 : 1 CB[10]⋅**AZAP** complex, some residual space inside the complex was found to be available and relevant for co‐guest inclusion. A small screening of planar (aromatic) guests converged toward monocationic co‐guests able to include in the 1 : 1 CB[10]⋅**AZAP** complex to afford heteroternary 1 : 1 : 1 host:guest:guest complexes with possibilities for planar dicationic co‐guests opened by **CG6**. These new heteroternary complexes could be used for the construction of advanced oligomeric structures or open new avenues toward new heteroguest pairs amenable for example, to supramolecular click binding with CB[10].

## Experimental Section

**Chemical compounds**: CB[10] was prepared according to a previous paper.^[37]^ **AZAP** was prepared according to the literature.^[38]^ *N,N,N*‐trimethyl‐benzenaminium iodide **CG1** was purchased from TCI and used without further purification. The synthesis of co‐guests **CG2** to **CG6** is described in Supporting Information.

**NMR titration of CB[10]⋅AZAP**: for each NMR tube, an amount of around 0.41 mg of CB[10] was precisely weighted and a precise volume of a 3 mM solution of **AZAP** was added to afford the selected CB[10]:**AZAP** ratio (between 1 : 8 to 1:0.125, Figure 2). The total volume (around 500 μL, depending on the weighted CB[10]) was adjusted with D_2_O to target 0.5 mM solutions of CB[10] (considering complete CB[10] solubility in D_2_O aided by the guest, even if CB[10] presents a very low solubility in D_2_O). Experimental results show that excess CB[10], even scarcely soluble without guest, plays a role in the titration.

**NMR titration of CB[10]⋅AZAP⋅CGx**: for each titration (**CG1**‐**CG6**), an amount of around 0.41 mg of CB[10] was weighted and a precise volume of a 3 mM solution of **AZAP** was added to afford the CB[10]:**AZAP** ratio 1:0.3. The total volume (∼500 μL, depending of weighted CB[10]) was adjusted with D_2_O to prepare a targeted 0.5 mM solution of CB[10]. Then corresponding volumes of stock solutions of **CGx** were added for the titrations shown on Figures S17–S22 of co‐guests **CG1‐CG6**, respectively.

## Conflict of interest

The authors declare no conflict of interest.

1

## Supporting information

As a service to our authors and readers, this journal provides supporting information supplied by the authors. Such materials are peer reviewed and may be re‐organized for online delivery, but are not copy‐edited or typeset. Technical support issues arising from supporting information (other than missing files) should be addressed to the authors.

Supporting Information

Supporting Information

Supporting Information

## Data Availability

The data that support the findings of this study are available from the corresponding author upon reasonable request.
