# Supplementary material for: Sequential Formation of Heteroternary Cucurbit[10]uril (CB[10]) Complexes
Source: Chemistry. 2022 Sep 14;28(64):e202201656. doi: 10.1002/chem.202201656 (PMC9826255; doi:10.1002/chem.202201656)
Supplement: Supplementary file 1 — Supporting Information [file CHEM-28-0-s003.pdf]

# Chemistry–A European Journal

Supporting Information


## Sequential Formation of Heteroternary Cucurbit[10]uril (CB[10]) Complexes


Chunyang Li, Anne-Doriane Manick, Yuxi Zhao, Fengbo Liu, Bastien Chatelet, Roselyne Rosas, Didier Siri, Didier Gigmes, Valerie Monnier, Laurence Charles, Julie Broggi, Simin Liu,* Alexandre Martinez,* Anthony Kermagoret,* and David Bardelang*

## Table of Contents

- **Experimental procedures**


1/ Chemical compounds ---------------------------------------------------------------------------------- S3

2/ NMR Measurements --------------------------------------------------------------------------------- S3

3/ Mass spectrometry ------------------------------------------------------------------------------------ S3

4/ Molecular modelling ------------------------------------------------------------------------------------ S4


- **Additional data**


5/ Syntheses and characterizations of **AZAP** and the coguests **CGx** ------------------------- S4

6/ $^{1}$H NMR spectra of CB[6], CB[7], CB[8] and CB[10] in $D_2O$ ---------------------------------- S5

7/ $^{1}$H NMR spectra of increased proportion of CB[10] with acetone in $D_2O$ ----------------- S6

8/ $^{1}$H NMR spectrum of CB[10] with acetone in $D_2O$ ---------------------------------------------- S6

9/ $^{1}$H NMR spectrum of the CB[10]•**AZAP** complex ---------------------------------------------- S7

10/ Distances from MD simulations of the CB[10]•**AZAP** complex ---------------------------- S7

11/ DOSY spectrum of the CB[10]•**AZAP** complex ----------------------------------------------- S8

12/ DOSY spectrum of the CB[10]•**AZAP•CG1** complex ---------------------------------------- S9

13/ DOSY spectrum of the CB[10]•**AZAP•CG2** complex ---------------------------------------- S9

14/ DOSY spectrum of the CB[10]•**AZAP•CG3** complex ---------------------------------------- S10

15/ DOSY spectrum of the CB[10]•**AZAP•CG4** complex ---------------------------------------- S10

16/ DOSY spectrum of the CB[10]•**AZAP•CG5** complex ---------------------------------------- S11

17/ $^{1}$H NMR titration of CB[10] with **CG2** ------------------------------------------------------------ S11

18/ $^{1}$H NMR titration of CB[10] with **CG6** ------------------------------------------------------------ S12

19/ $^{1}$H NMR titration of CB[10]•**AZAP** with addition of **CG2** ------------------------------------ S12

20/ Accurate mass measurements of heteroternary complexes -------------------------------- S13

21/ $^{1}$H NMR titration and Job Plot for the CB[10]•**AZAP** complex with **CG2** ----------------- S13

22/ $^{1}$H NMR titration of the CB[10]•**AZAP** complex with **CG1** --------------------------------- S14

23/ $^{1}$H NMR titration of the CB[10]•**AZAP** complex with **CG2** --------------------------------- S14

24/ $^{1}$H NMR titration of the CB[10]•**AZAP** complex with **CG3** ---------------------------------- S15
25/ $^{1}$H NMR titration of the CB[10]•**AZAP** complex with **CG4** ---------------------------------- S15
26/ $^{1}$H NMR titration of the CB[10]•**AZAP** complex with **CG5** ---------------------------------- S16
27/ $^{1}$H NMR titration of the CB[10]•**AZAP** complex with **CG6** ---------------------------------- S16
28/ Control from the mixture of solutions of CB[10]•**AZAP** and CB[10]•**CG3** --------------- S17
29/ Preliminary crystal structures --------------------------------------------------------------------- S18
30/ Snapshots of the CB[10]•**AZAP•CG5** complex from the corresponding MD trajectory S19
31/ Distances between the barycenter of molecules from MD simulations ------------------- S20
32/ References ------------------------------------------------------------------------------------------ S26

## - Experimental procedures

***1/ Chemical compounds.*** N,N,N-trimethyl-benzenaminium iodide, imidazo[1,5-a]pyridine, iodomethane, 1-methylbenzimidazole, quinoline, isoquinoline, 1,4-bis(bromomethyl)-benzene, trimethylamine solvents (DMSO-D6, $D_2O$, acetonitrile, tetrahydrofuran, diethyl ether) were purchased from Aldrich, Acros or TCI and used without further purification. CB[10] was prepared according to a previous paper.[1] **AZAP** was prepared according to the literature.[2] N,N,N-trimethyl-benzenaminium iodide **CG1** was purchased from TCI and used without further purification. The synthesis of coguests **CG2** to **CG6** is described hereafter.

***2/ NMR Measurements.*** NMR spectra were recorded on BRUKER Avance III nanobay – 300 or 400 spectrometers, a BRUKER Avance HD – 500 spectrometer and a BRUKER Avance III 600 MHz spectrometer ($^1$H-NMR frequencies 300.13, 400.13, 500.13 and 600.13 MHz respectively) at 300 K using $D_2O$ as the solvent and a watergate sequence (water suppress) when necessary (potentially affecting signals and integrals near the signal suppressed). Acetone was also used when necessary as a reference. Splitting patterns are indicated as follows: s, singlet; d, doublet; t, triplet; m, multiplet. 2D DOSY experiments were acquired on the 500 MHz spectrometer equipped with a multi-nuclei 5mm BBFO probe with Z-gradient using Bruker Topspin 3.6.2 software, a pulse sequence that incorporated bipolar gradient pulses and a longitudinal eddy current delay (LED). A total of 16 gradient values were linearly sampled from 6% to 95%. 32 scans were acquired with 32k data points, for a total acquisition time of ca. 35 min. The gradient pulse duration ( $\partial$ /2) and the diffusion time ($\Delta$) were set to 1.7 ms and 80 ms, respectively, with a gradient recovery delay of 0.1 ms and a LED of 5 ms. The DOSY spectra were simply obtained by using the single-exponential fitting routine of the EDDOSY feature of the TopSpin software from Bruker.

*NMR titration of **CB[10]•AZAP***: for each NMR tube, an amount of around 0.41 mg of CB[10] was precisely weighted and a precise volume of a 3 mM solution of **AZAP** was added to afford the selected CB[10]:**AZAP** ratio (between 1:8 to 1:0.125, Figure 2). The total volume (around 500 µL, depending on the weighted CB[10]) was adjusted with $D_2O$ to target 0.5 mM solutions of CB[10] (considering complete CB[10] solubility in $D_2O$, even if CB[10] presents a very low solubility in $D_2O$). Experimental results show that excess CB[10], even if scarcely soluble without guest, plays a role in the titration.

*NMR solutions for DOSY (Figure 4 and Figures S4-S9):* an amount of around 0.41 mg of CB[10] was weighted and volumes of a 3 mM solution of **AZAP** and of a 10 mM solution of **CGx** were added to afford a 1:0.3:0.3 **CB[10]•AZAP•CGx**_ratio._The total volume (around 500 µL, depending of the weighted CB[10]) was adjusted with $D_2O$ to prepare a targeted 0.5 mM solution of CB[10].

*NMR titration of **CB[10]•AZAP•CGx:*** for each titration (**CG1**-**CG6**), an amount of around 0.41 mg of CB[10] was weighted and a precise volume of a 3 mM solution of **AZAP** was added to afford the CB[10]:**AZAP** ratio 1:0.3. The total volume (~500 µL, depending of weighted CB[10]) was adjusted with $D_2O$ to prepare a targeted 0.5 mM solution of CB[10]. Then corresponding volumes of stock solutions of **CGx** were added for the titrations in Figures S13-S18 of coguests **CG1-CG6**, respectively.

***3/ Mass spectrometry.*** High resolution mass spectrometry experiments were performed with a Synapt G2 HDMS mass spectrometer (Waters, Manchester, UK) equipped with a pneumatically assisted electrospray ionization (ESI) source operated at 35°C in the positive ion mode (electrospray voltage: +2.8 kV; declustering potential: +20 V; nebulizing gas flow: $N_2$, 100 L.h$^{-1}$). Mass spectra were acquired using an orthogonal acceleration time-of-flight (oa-TOF) mass analyzer. The sample was diluted (1/10) in methanol then injected in the ESI source at a 10 µL.min$^{-1}$ flow rate using a syringe pump. Accurate mass measurements were achieved using an external calibration for the oa-TOF mass analyzer. Instrument control, data acquisition and data processing were performed with the MassLynx 4.1 programs provided by Waters.

***4/ Molecular modelling.*** All Molecular Dynamics calculations were performed with Gromacs 2021 software.[3] The compounds were embedded in a cubic box containing TIP3P[4] water molecules. After an energy minimization to avoid close contacts, we relaxed the volume of the simulation box by performing an NPT simulation of 300 ps duration (300 K, 1 atm, time step of 0.25 fs). Chlorides were used as counter-ions. Once this initial run was achieved, the simulations were then restarted in the NVT ensemble during 100 ns (300 K, time step of 0.25 fs) using GAFF force field with RESP[5] atomic charges. The barycenter of CB[10] was calculated as the center of gravity of the 20 central carbon atoms of the glycoluril moieties.

## - Additional data

***5/ Syntheses and characterizations of AZAP and the coguests CG1 to CG6.***

*N,N,N-trimethyl-benzenaminium iodide **CG1** (CAS: 98-04-4)*

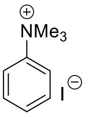

**CG1**

N,N,N-trimethyl-benzenaminium iodide was purchased from TCI and used without further purification.

*Synthesis of 2-methylimidazo[1,5-a]pyridin-2-ium iodide **CG2** (CAS: 23879-76-7)*

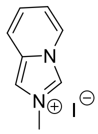

**CG2**

To a solution of imidazo[1,5-a]pyridine (1.0 g, 8.4 mmol, 1.0 equiv) in acetonitrile (15 ml) was added iodomethane (57.0 µL, 9.2 mmol, 1.1 equiv) at room temperature. The mixture was refluxed at 90°C in a sealed flask for 16 hours. After cooling to room temperature, diethyl ether (15 mL) was added. The precipitate was filtered and washed with diethyl ether (3x15 mL). The resulting solid was dried under vacuum for 24 hours to give 2-methylimidazo[1,5-a]pyridin-2-ium iodide **CG2** as a beige solid (2 g, >99%). $^1$H NMR (250 MHz, DMSO-*d*$_6$) δ (ppm): 4.17 (s, 3H, $NCH_3$), 7.22 (dd, *J* = 7 and 9 Hz, 2H, 2H$^{Ar}$), 7.85 (d, *J* = 9 Hz, 1H, H$^{Ar}$), 8.18 (s, 1H, H$^{Ar}$), 8.60 (d, *J* = 7 Hz, 1H, H$^{Ar}$), 9.65 (s, 1H, H$^{Ar}$). The $^1$H NMR spectrum was consistent with the literature.[6] HRMS (ESI) *m/z* : Calcd for $C_8H_9N_2^+$ 133.0760; Found 133.0761.

*Synthesis of 1,3-dimethylbenzimidazolium iodide **CG3** (CAS: 7181-87-5)*

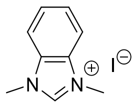

**CG3**

To a solution of 1-methylbenzimidazole (3.0 g, 22.7 mmol, 1.0 equiv.) in anhydrous acetonitrile (15 mL) was added iodomethane (2.2 mL, 34.05 mmol, 1.5 equiv.). The mixture was stirred at 45°C for 24 h. Diethyl ether (50 mL) was added to the suspension to facilitate the precipitation of the solid. The latter was collected by filtration, washed with diethyl ether (3 x 50 mL) and dried under vacuum to give 1,3-dimethylbenzimidazolium iodide ***CG3*** (5.29 g, 85%) as white needles. $^1$H NMR: (300 MHz, DMSO-*d*$_6$) δ 4.08 (s, 6H, $NCH_3$), 7.70 (dd, *J* = 3 and 6.3 Hz, 2H, 2H$^{Ar}$), 8.02 (dd, *J* = 3 and 6.3 Hz, 2H, 2H$^{Ar}$), 9.67 (s, 1H, CH=N$^+$). The $^1$H NMR spectrum was consistent with the literature.[7] HRMS (ESI) *m/z* : Calcd for $C_9H_{11}N_2^+$ 147.0917; Found 147.0914.

*Synthesis of 1-methylquinolin-1-ium iodide **CG4** (CAS: 3947-76-0)*

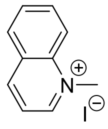

**CG4**

In a sealed flask, quinoline (5 g, 38.7 mmol, 1 equiv.) and iodomethane (7.3 mL, 116.14 mmol, 3.0 equiv.) were dissolved in anhydrous acetonitrile and stirred at 90°C for 24 h. After cooling to room temperature, diethyl ether (50 mL) was added to the suspension to facilitate the precipitation of the solid. The latter was filtered, washed with diethyl ether (3 x 100 mL) and dried under vacuum to give 1-methylquinolin-1-ium iodide **CG4** (10.3 g, 98%) as a yellow solid. $^1$H NMR: (400 MHz, DMSO-*d*$_6$) δ 4.64 (s, 3H, $NCH_3$), 8.07 (dt, *J* = 0.4 and 8 Hz, 1H, H$^{Ar}$), 8.17 (dd, *J* = 5.6 and 8.4 Hz, 1H, H$^{Ar}$), 8.30 (dt, *J* = 1.6 and 7.2 Hz, 1H, H$^{Ar}$), 8.47-8.53 (m, 2H, H$^{Ar}$), 9.28 (d, *J* = 8.4 Hz, 1H, H$^{Ar}$), 9.50 (d, *J* = 5.6 Hz, 1H, H$^{Ar}$). The $^1$H NMR spectrum was consistent with the literature.[8] HRMS (ESI) *m/z* : Calcd for $C_{10}H_{10}N^+$ 144.0808; Found 144.0812.

*Synthesis of 2-methylisoquinolin-2-ium iodide **CG5** (CAS: 3947-77-1)*

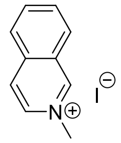

CG5

Iodomethane (2.637g, 18.58 mmol, 1.2 equiv.) was added to a solution of isoquinoline (2g, 15.5 mmol, 1.0 equiv.) in anhydrous tetrahydrofuran (5.0 mL). The solution was stirred at room temperature for 24 h. The precipitate was collected by filtration and washed with ethyl acetate (200 mL) and diethyl ether (200 mL). The solid was dried under vacuum to give 2-methylisoquinolin-2-ium iodide **CG5** (3.183g, 76%) as a pale yellow powder. $^{1}$H NMR: (400 MHz, DMSO-$d_6$) δ 4.49 (s, 3H, $N^{+}CH_3$), 8.06 (t, $J$ = 7.6 Hz, 1H, $H^{Ar}$), 8.23 (t, $J$ = 8.0 Hz, 1H, $H^{Ar}$), 8.34 (d, $J$ = 8.4 Hz, 1H, $H^{Ar}$), 8.47 (d, $J$ = 8.0 Hz, 1H, $H^{Ar}$), 8.58 (d, $J$ = 6.8 Hz, 1H, $H^{Ar}$), 8.71 (d, $J$ = 6.8 Hz, 1H, $H^{Ar}$), 10.07 (s, 1H, $H^{Ar}$). The $^{1}$H NMR spectrum was consistent with the literature.[9] HRMS (ESI) *m/z* : Calcd for $C_{10}H_{10}N^{+}$ 144.0808; Found 144.0809.

*Synthesis of (p-phenylenedimethylene)bis[trimethylammonium] bromide **CG6** (CAS: 16846-81-4)*

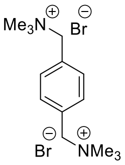

CG6

*(p-phenylenedimethylene)bis[trimethylammonium]* ***PMTA*** *was synthesized by reaction of* 1,4-bis(bromomethyl)-benzene (2.95 g, 11.2 mmol) with trimethylamine (15 ml in 25% solution in water, 57 mmol) in 30 mL of ethanol at 40 °C for 24 h. The resulting precipitate was filtered, washed with acetone before drying to yield **CG6** as a white power (2.15 g, 50% yield). $^{1}$H NMR (300 MHz, $D_2O$) δ 7.72 (s, 4H), 4.58 (s, 4H), 3.15 (s, 18H), consistent with literature.[10] HRMS (ESI) *m/z* : Calcd for $C_{14}H_{26}N_2^{2+}$ 111.1043; Found 111.1040.

### *6/ $^{1}$H NMR spectra of CB[6], CB[7], CB[8] and CB[10] in $D_2O$.*

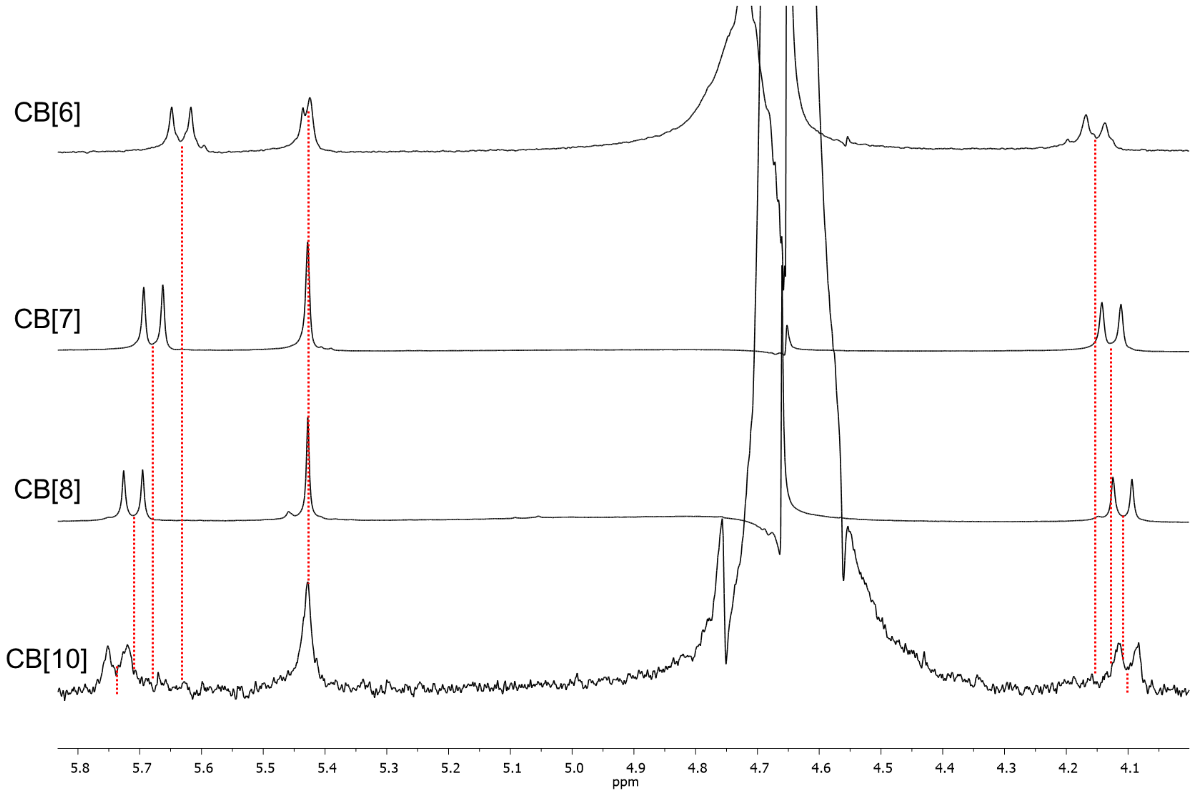


***Figure S1***. $^{1}$H NMR spectra of CB[6], CB[7], CB[8] and CB[10] in $D_2O$.

***7/ ¹H NMR spectra of increased proportion of CB[10] with acetone in D₂O.***

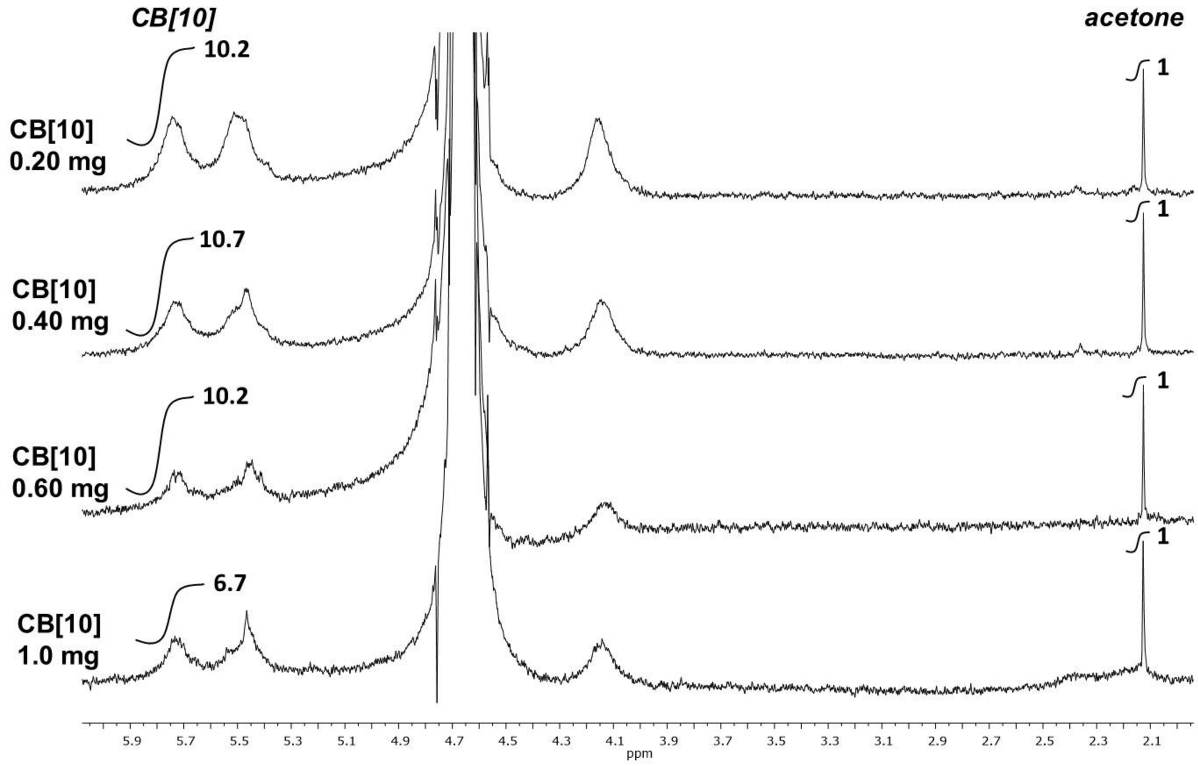


***Figure S2***. ¹H NMR spectra of increased proportion of CB[10] with acetone in $D_2O$.

***8/ ¹H NMR spectrum of CB[10] with acetone in D₂O.***

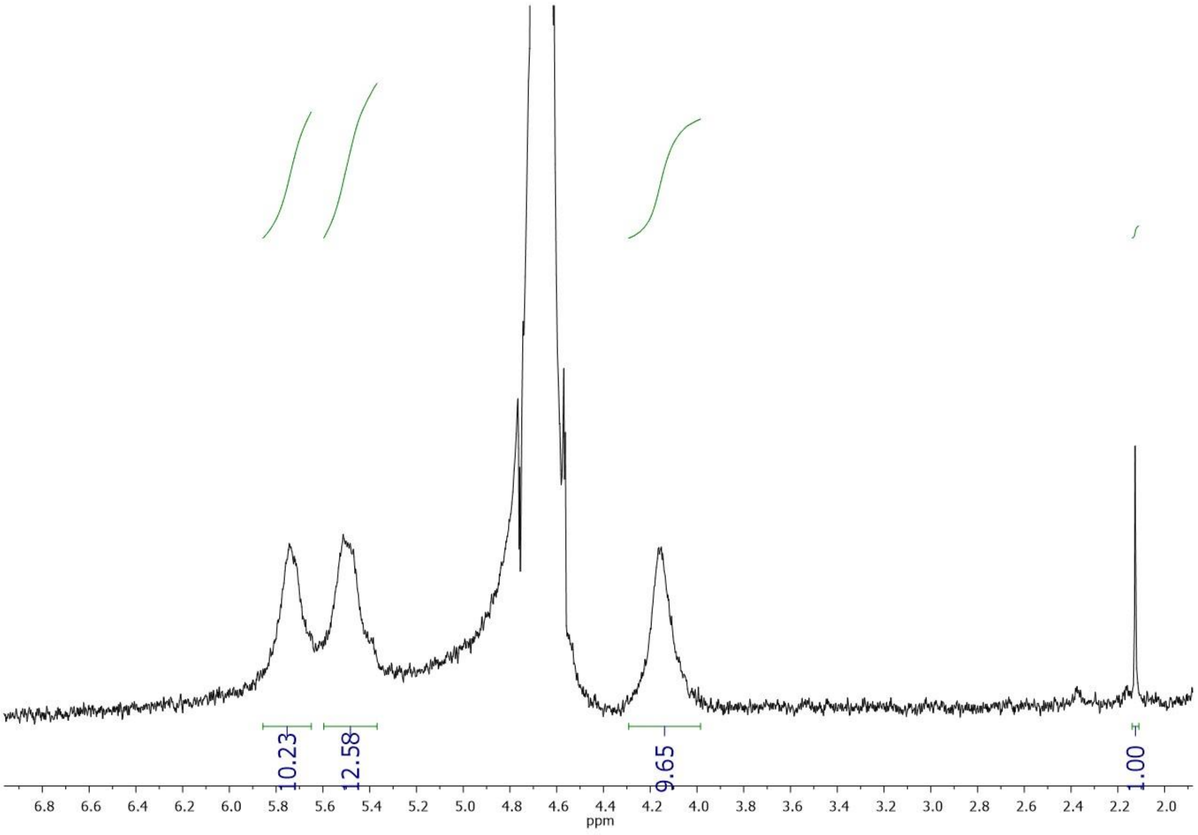


***Figure S3***. ¹H NMR spectrum of CB[10] (0.2 mg) in $D_2O$ with a controlled volume of acetone.

### *9/ ¹H NMR spectrum of the CB[10]•AZAP complex*

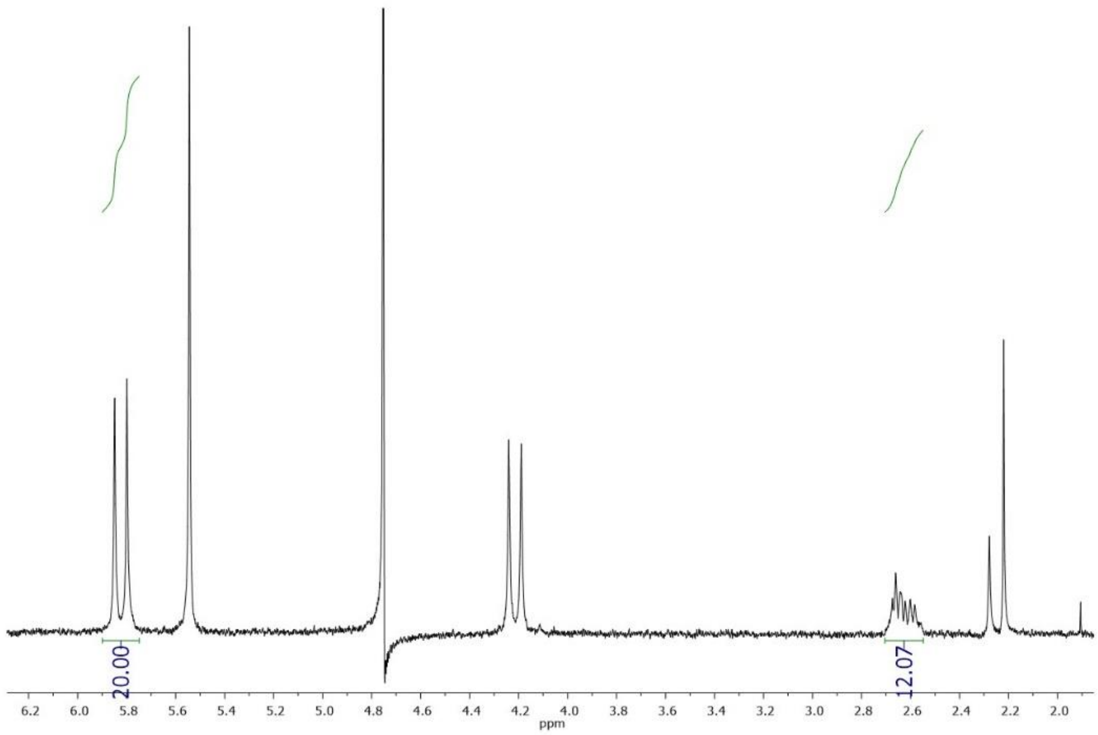


***Figure S4***. $^1$H NMR spectrum of the CB[10]•**AZAP** complex in $D_2O$ (500 MHz, Ref acetone 2.22 ppm, 0.125 eq. **AZAP**, ratio of integrals for host and guest protons is consistent with a 1:1 complex).

### *10/ Distances from MD simulations of the CB[10]•AZAP complex*

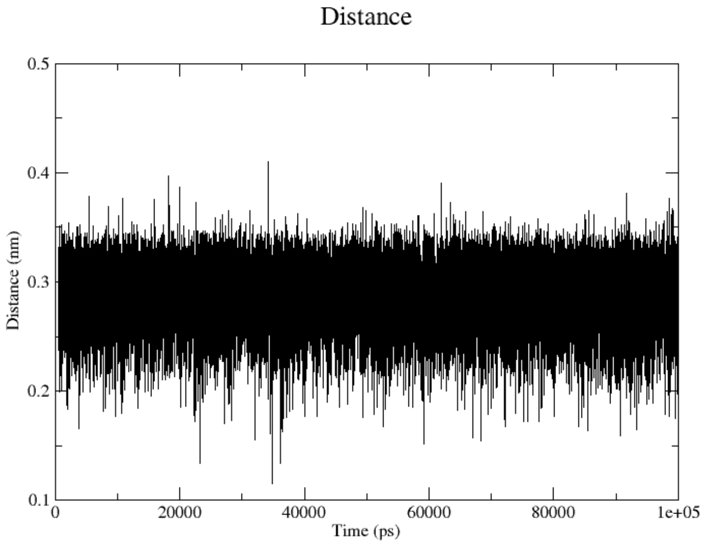


***Figure S5***. Distance between the barycentre of **AZAP** and the barycentre of CB[10].

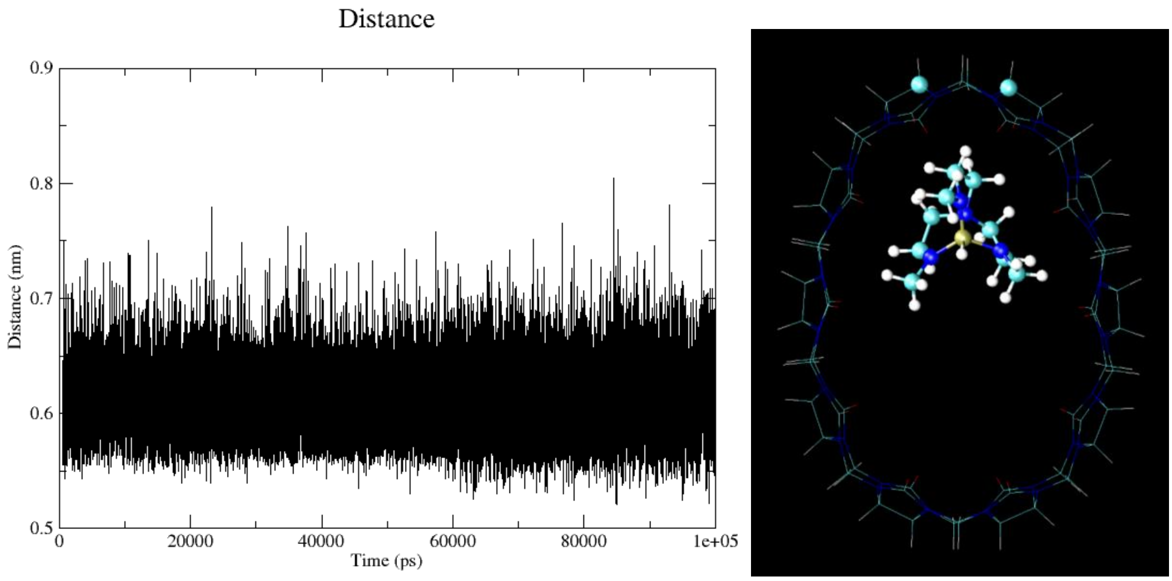


***Figure S6***. Distance between the barycentre of **AZAP** and the barycentre of the two carbon atoms of CB[10] highlighted on the Figure on the right, illustrating the trend for **AZAP** to sit in one part of the cavity while occasionally traveling through it.

### *11/ DOSY spectrum of the CB[10]•AZAP complex*

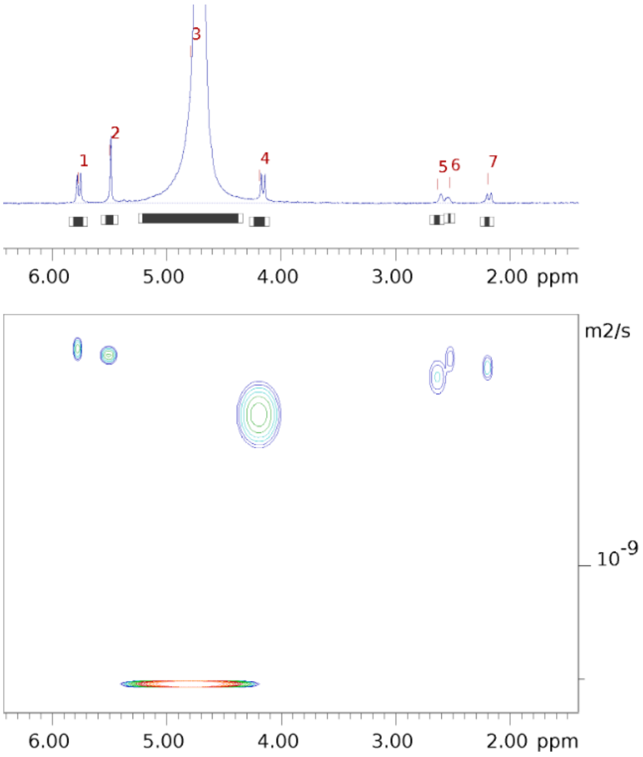


***Figure S7***. DOSY NMR spectrum of the CB[10]•**AZAP** complex in water. Numbers correspond to portions of spectrum taken into account for tentative calculations of diffusion coefficients.

*12/ DOSY spectrum of the CB[10]•AZAP•CG1 complex*

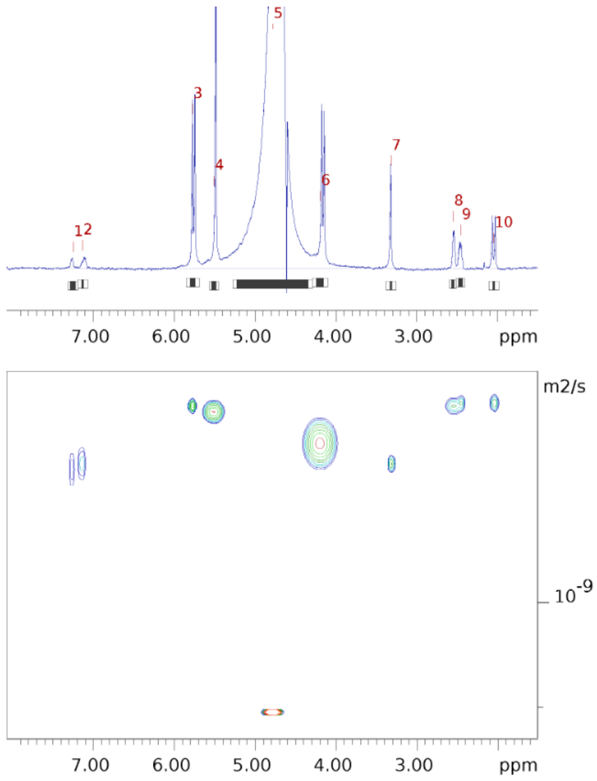


***Figure S8***. DOSY NMR spectrum of the CB[10]•**AZAP•CG1** complex in water. Numbers correspond to portions of spectrum taken into account for tentative calculations of diffusion coefficients.

*13/ DOSY spectrum of the CB[10]•AZAP•CG2 complex*

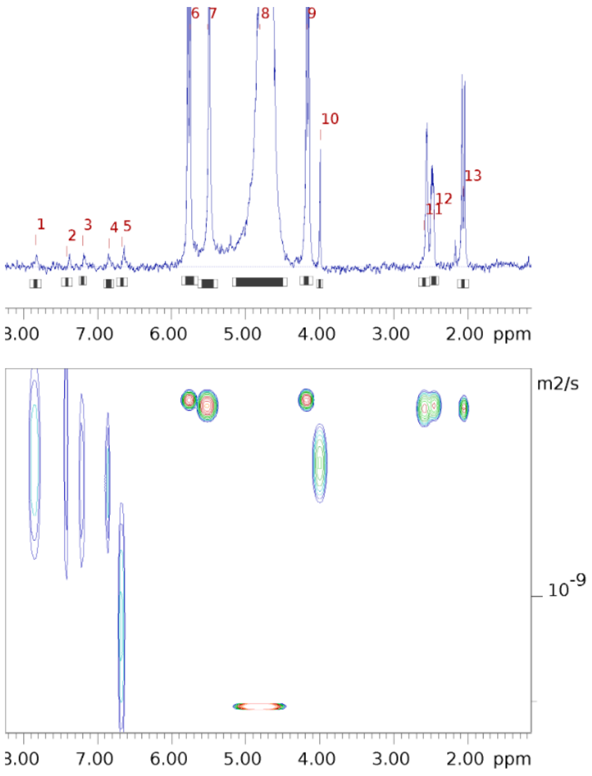


***Figure S9***. DOSY NMR spectrum of the CB[10]•**AZAP•CG2** complex in water. Numbers correspond to portions of spectrum taken into account for tentative calculations of diffusion coefficients.

### *14/ DOSY spectrum of the CB[10]•AZAP•CG3 complex*

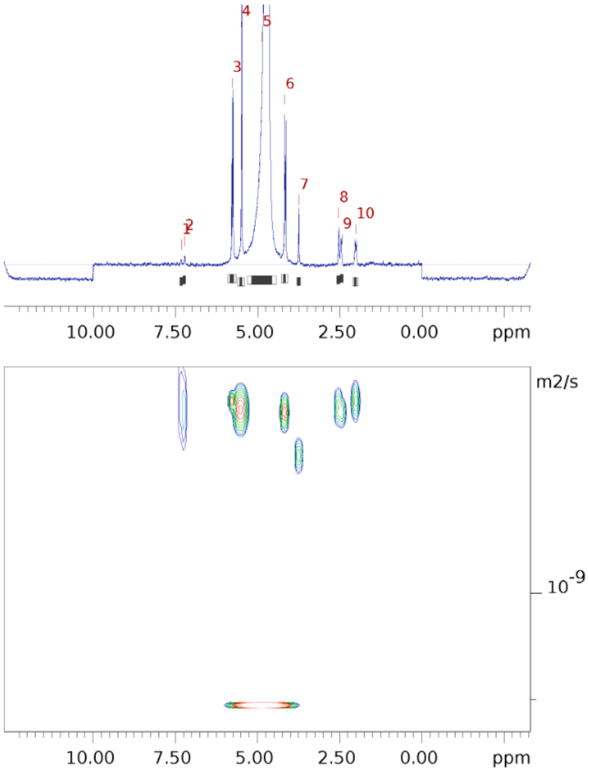


***Figure S10***. DOSY NMR spectrum of the CB[10]•**AZAP•CG3** complex in water. Numbers correspond to portions of spectrum taken into account for tentative calculations of diffusion coefficients.

### *15/ DOSY spectrum of the CB[10]•AZAP•CG4 complex*

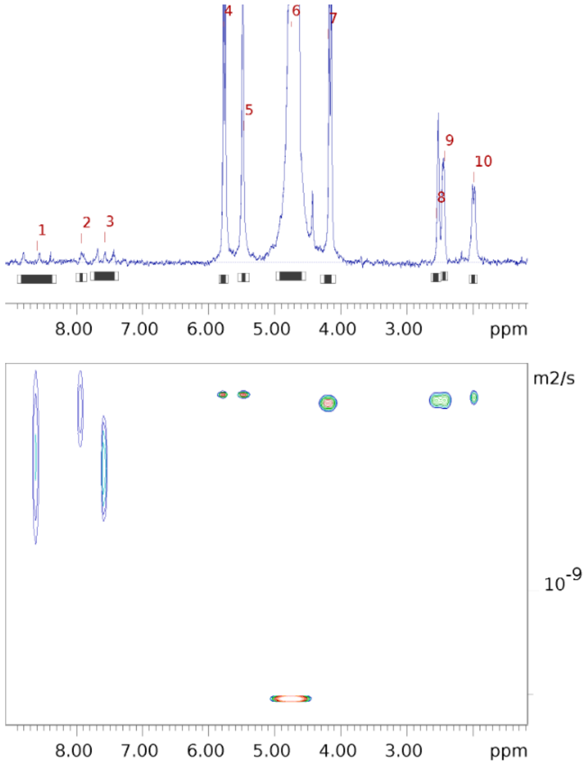


***Figure S11***. DOSY NMR spectrum of the CB[10]•**AZAP•CG4** complex in water. Numbers correspond to portions of spectrum taken into account for tentative calculations of diffusion coefficients.

## 16/ DOSY spectrum of the CB[10]•AZAP•CG5 complex

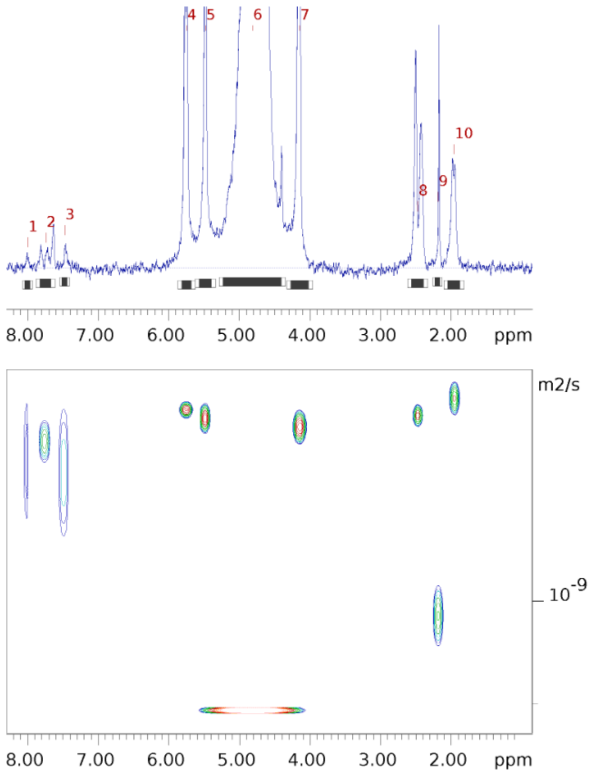


***Figure S12***. DOSY NMR spectrum of the CB[10]•**AZAP•CG5** complex in water. Numbers correspond to portions of spectrum taken into account for tentative calculations of diffusion coefficients.

## *17/ $^1$H NMR titration of CB[10] with CG2*

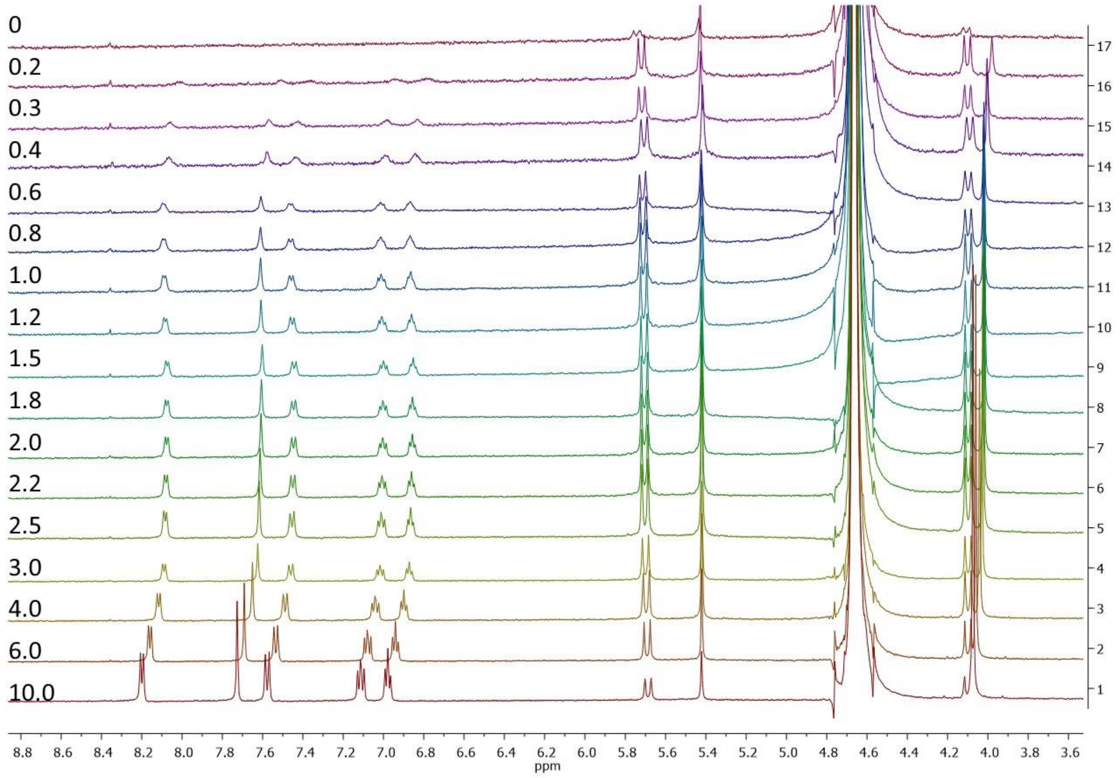


***Figure S13***. $^1$H NMR titration of CB[10] (constant) with **CG2**.

### *18/ $^{1}$H NMR titration of CB[10] with CG6*

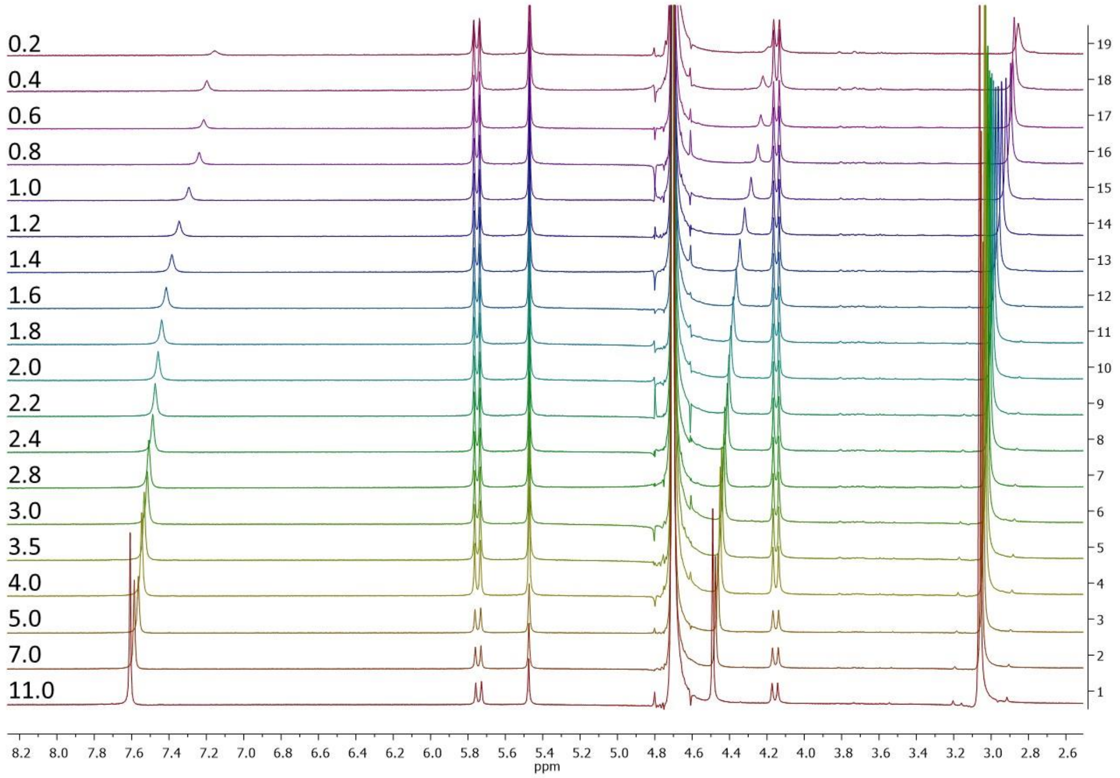


***Figure S14***. $^{1}$H NMR titration of CB[10] (constant) with **CG6**.

### *19/ $^{1}$H NMR titration of CB[10]•AZAP with addition of CG2.*

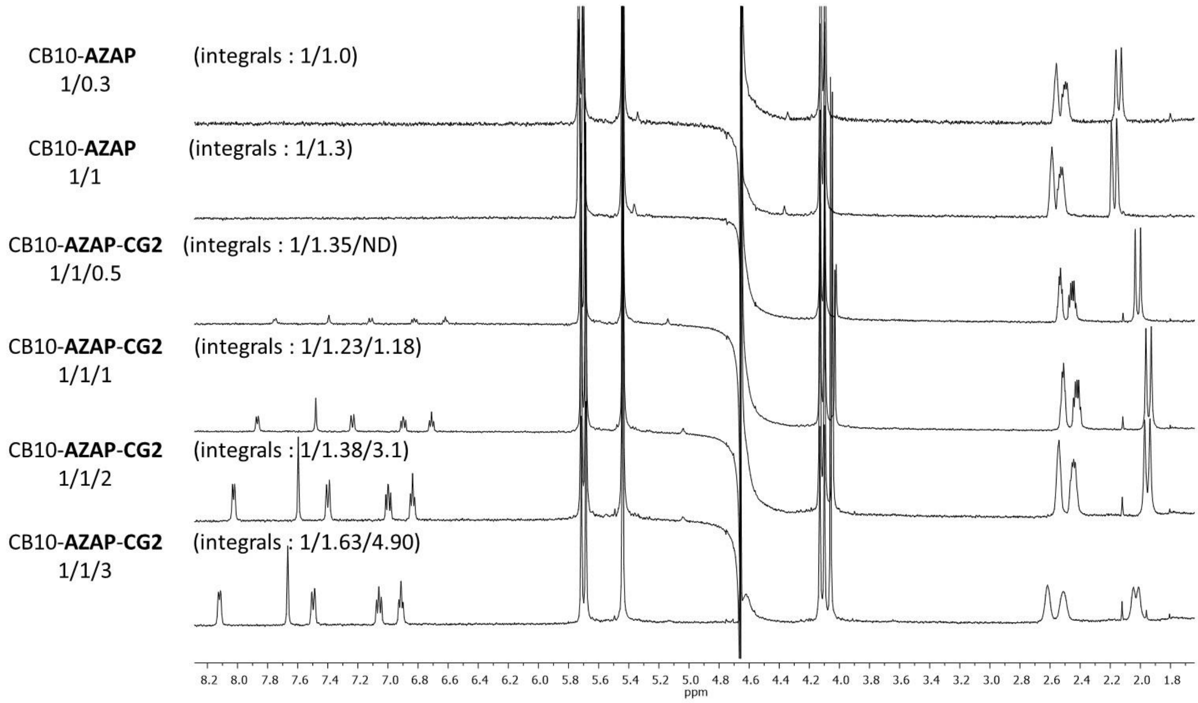


***Figure S15***. $^{1}$H NMR titration of CB[10]•**AZAP** with addition of **CG2**.

***20/ Accurate mass measurements of heteroternary complexes***

CB[10]•**AZAP•CG1**
HRMS (ESI) *m/z* : Calcd for $C_{78}H_{96}N_{45}O_{20}P^{2+}$ 1006.8802; Found 1006.8804
CB[10]•**AZAP•CG2**
HRMS (ESI) *m/z* : Calcd for $C_{77}H_{91}N_{46}O_{20}P^{2+}$ 1005.3622; Found 1005.3620
CB[10]•**AZAP•CG3**
HRMS (ESI) *m/z* : Calcd for $C_{78}H_{93}N_{46}O_{20}P^{2+}$ 1012.3700; Found 1012.3712
CB[10]•**AZAP•CG4**
HRMS (ESI) *m/z* : Calcd for $C_{79}H_{92}N_{45}O_{20}P^{2+}$ 1010.8646; Found 1010.8641
CB[10]•**AZAP•CG5**
HRMS (ESI) *m/z* : Calcd for $C_{79}H_{92}N_{45}O_{20}P^{2+}$ 1010.8646; Found 1010.8648

***21/ $^{1}$H NMR titration and Job Plot for the CB[10]•AZAP complex with CG2***

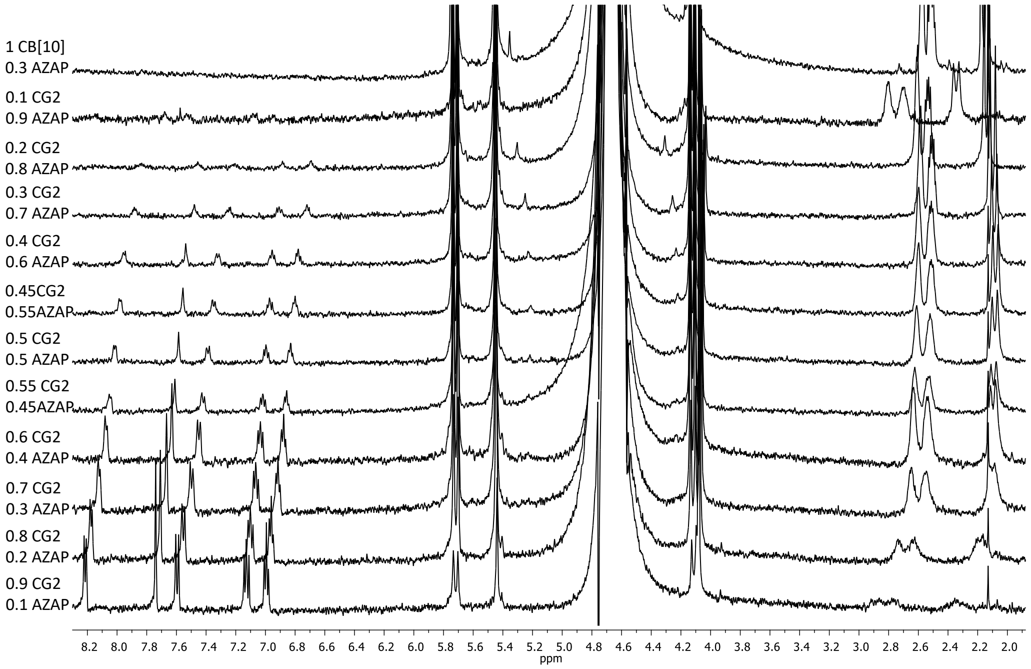


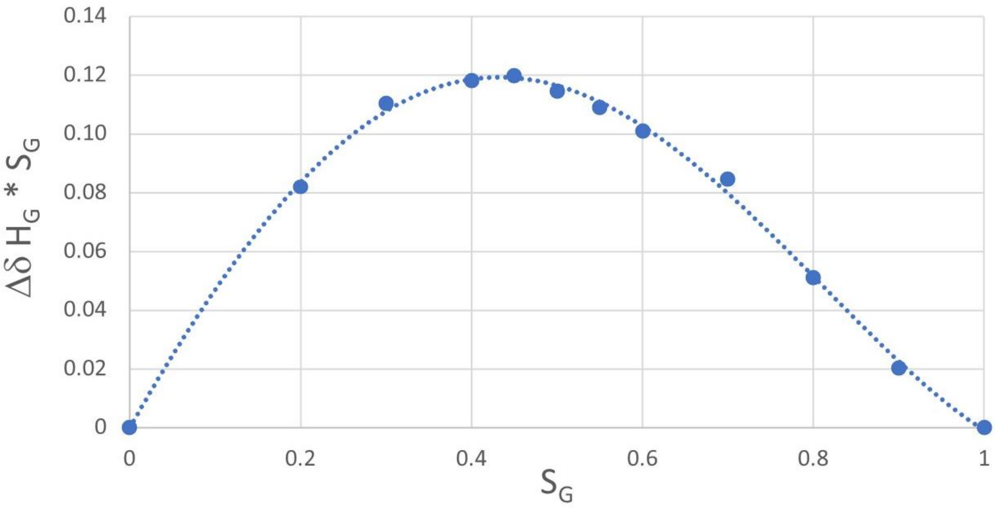


***Figure S16***. NMR and Job Plot for the CB[10]•**AZAP** complex with **CG2** in $D_2O$. All job plots considering protons of **CG2** are identical. They all show a maximum of the bell-shape curve at 0.45, near 0.5 the value expected for a 1:1 (CB[10]•**AZAP**:**CG2**) stoichiometry.

## 22/ ¹H NMR titration of the CB[10]•AZAP complex with CG1

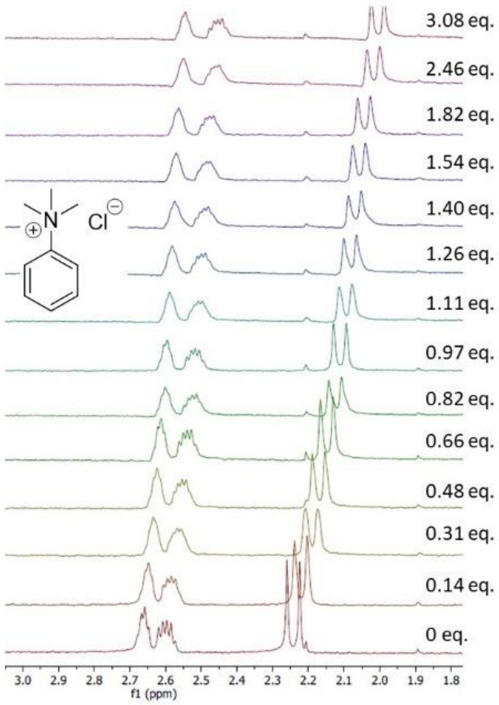


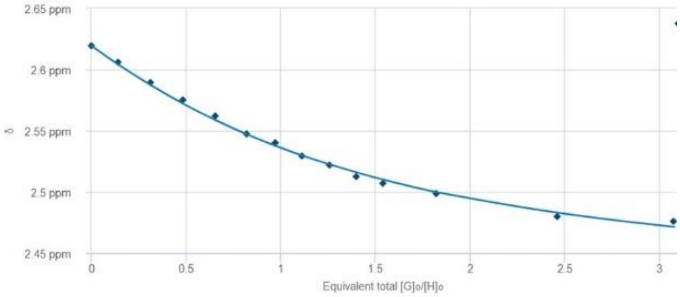

| Parameter (bounds) | Optimised | Error |
|---|---|---|
| K ( 0 → ∞ ) | 6768.46 $M^{-1}$ | ± 4.8100 % |

***Figure S17***. ¹H NMR spectra of the CB[10]•**AZAP** complex with **CG1** and $K_a$ calculation.

## 23/ ¹H NMR titration of the CB[10]•AZAP complex with CG2

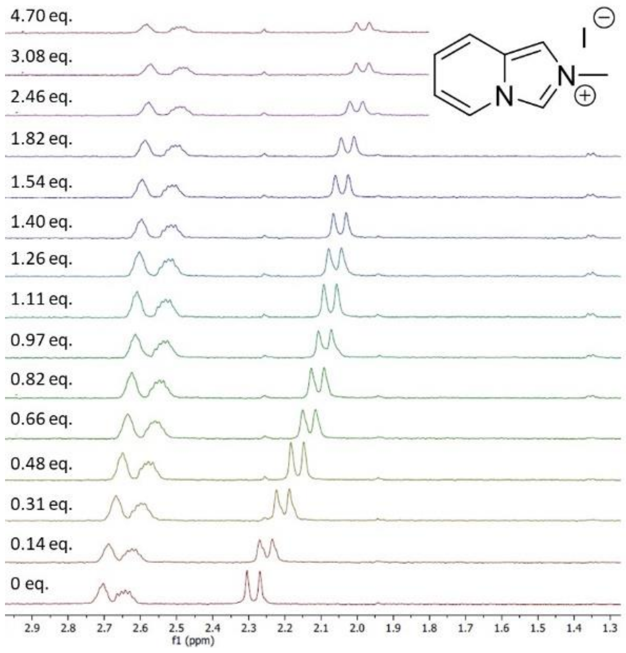


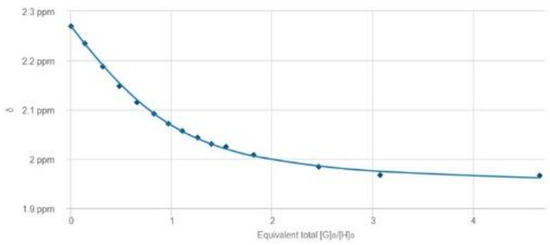

| Parameter (bounds) | Optimised | Error |
|---|---|---|
| K ( 0 → ∞ ) | 25100.48 $M^{-1}$ | ± 5.4934 % |

***Figure S18***. ¹H NMR spectra of the CB[10]•**AZAP** complex with **CG2** and $K_a$ calculation.

## 24/ $^{1}$H NMR titration of the CB[10]•AZAP complex with CG3

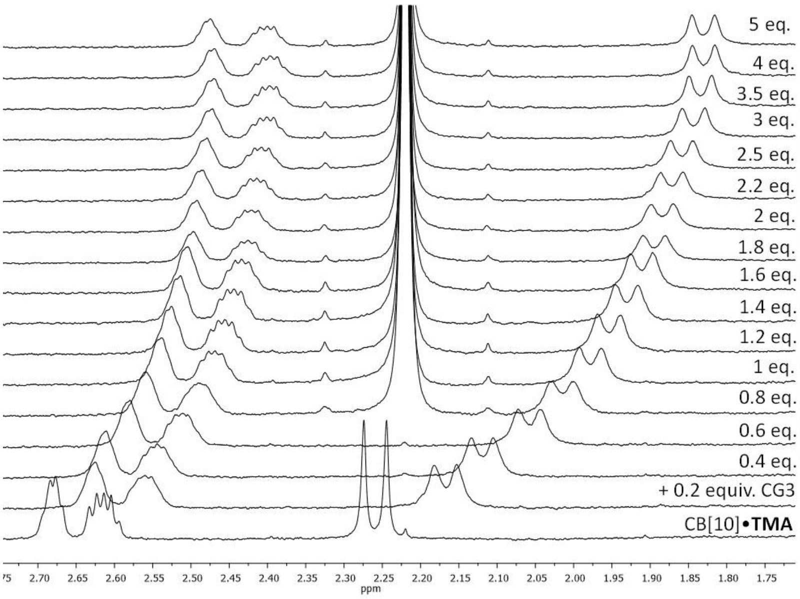


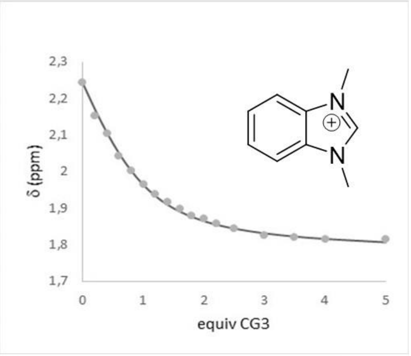


| K (M-1) | K error (%) |
|---|---|
| 25020 | 6,6 |

***Figure S19***. $^{1}$H NMR spectra of the CB[10]•**AZAP** complex with **CG3** and $K_a$ calculation.

## 25/ $^{1}$H NMR titration of the CB[10]•AZAP complex with CG4

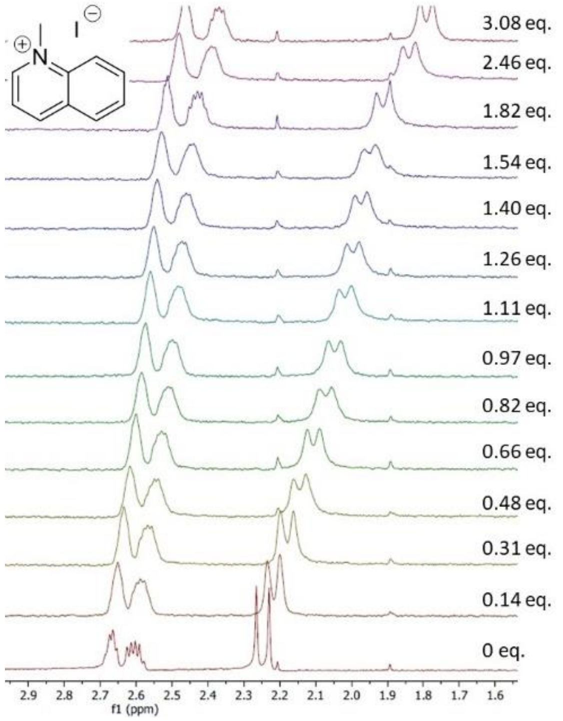


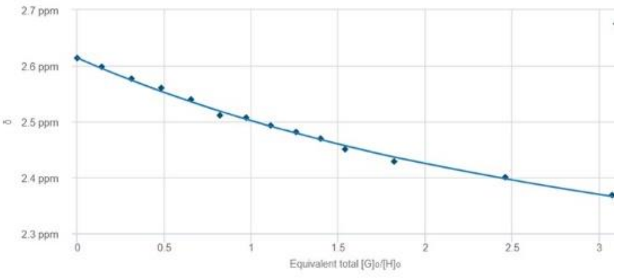


| Parameter (bounds) | Optimised | Error |
|---|---|---|
| **K** ( 0 → ∞ ) | 1509.10 M⁻¹ | ± 3.1935 % |

***Figure S20***. $^{1}$H NMR spectra of the CB[10]•**AZAP** complex with **CG4** and $K_a$ calculation.

### *26/ $^{1}$H NMR titration of the CB[10]•AZAP complex with CG5*

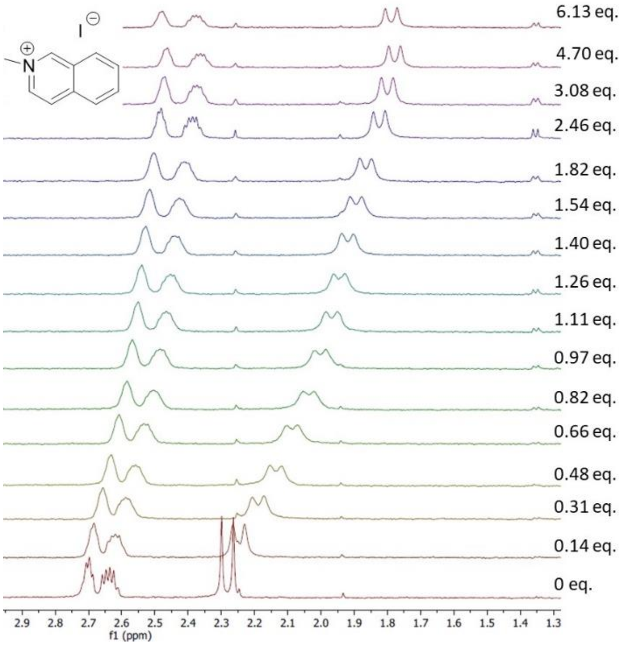


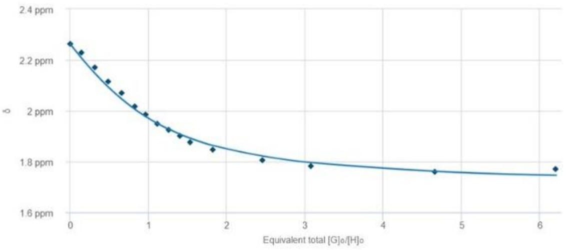


| Parameter (bounds) | Optimised | Error |
|---|---|---|
| **K** ( 0 → ∞ ) | 13624.54 $M^{-1}$ | ± 11.2602 % |

***Figure S21***. $^{1}$H NMR spectra of the CB[10]•**AZAP** complex with **CG5** and $K_a$ calculation.

### *27/ $^{1}$H NMR titration of the CB[10]•AZAP complex with CG6*

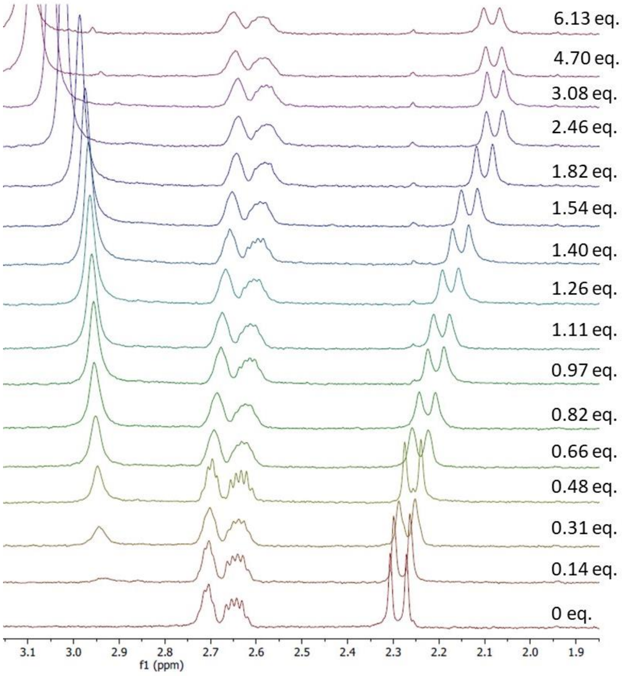


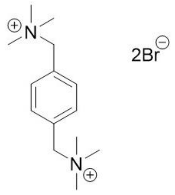


**CG6**

***Figure S22***. $^{1}$H NMR spectra of the CB[10]•**AZAP** complex with **CG6**.

A slight deviation from the curve expected for a 1:1 binding was observed preventing the calculation of the binding constant corresponding to the equilibrium: CB[10]•**AZAP**+**CG6** ⇆ CB[10]•**AZAP**•**CG6**.

***28/ Control from the mixture of solutions of CB[10]•AZAP and CB[10]•CG3.***

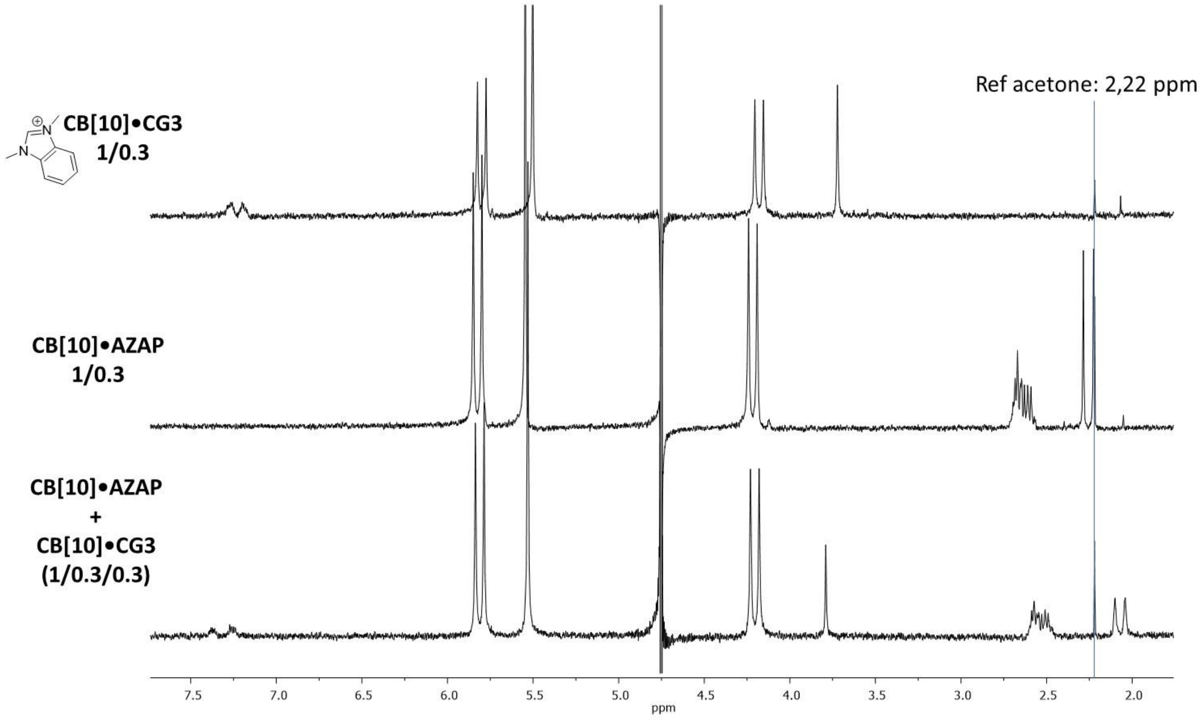


***Figure S23***. $^{1}$H NMR spectrum resulting from the mixture of solutions of CB[10]•**AZAP** and CB[10]•**CG3** and in line with formation of the new heteroternary complex CB[10]•**AZAP**•**CG3**.

***29/ Preliminary crystal structures***

CB[10] structure crystallized from a solution of CB[10]+**AZAP**

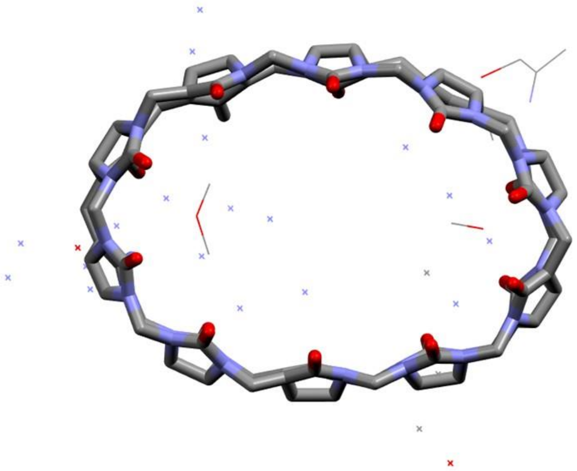

CB[10] structure crystallized from a solution of CB[10]+**AZAP**+**CG2**

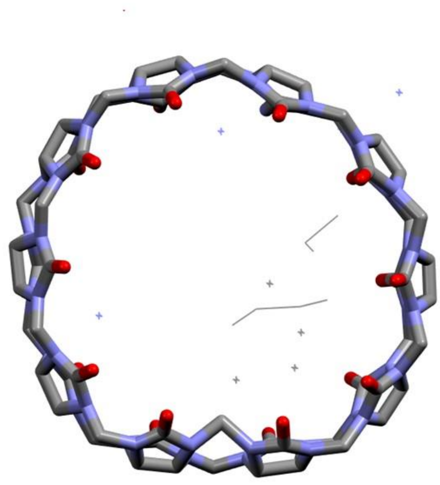

***Figure S24***. Preliminary crystal structures of CB[10] from (top) a solution of CB[10] and **AZAP** and (bottom) a solution of CB[10], **AZAP** and **CG2**.

These structures should be considered with extreme caution due to the high solvent and guest disordering which prevented us from finalizing the refinement.

***30/ Snapshots of the CB[10]•AZAP•CG5 complex from the corresponding MD trajectory***

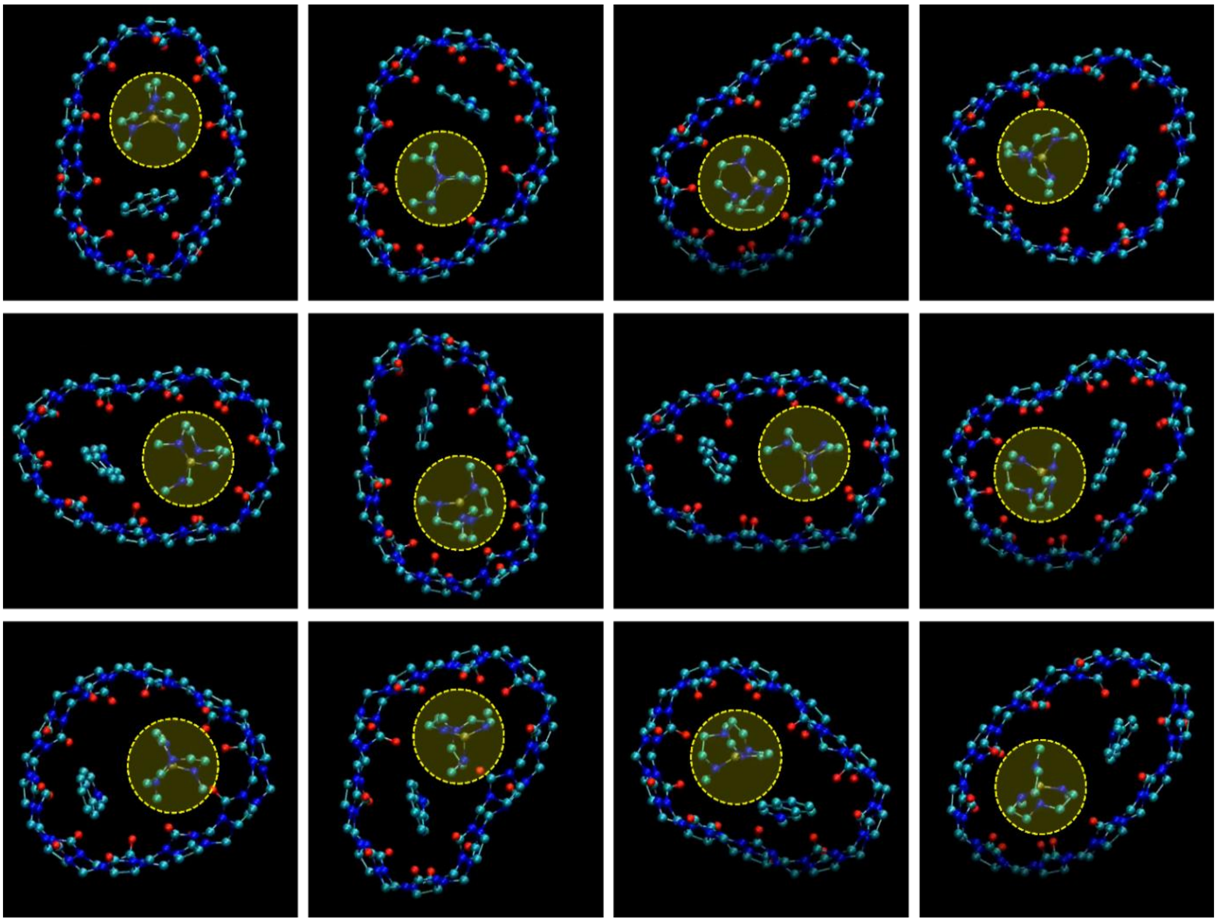

***Figure S25***. snapshots of the CB[10]•**AZAP•CG5** complex from the corresponding molecular dynamics trajectory in water. Note the high mobility of **AZAP**, of coguest **CG5** and the adaptability of the CB[10] macrocycle to wrap around the two included molecules (see text and video S2; the yellow disk delineated by a yellow dashed line denotes the AZAP location in each case).

***31/ Distances between the barycenter of molecules from MD simulations***

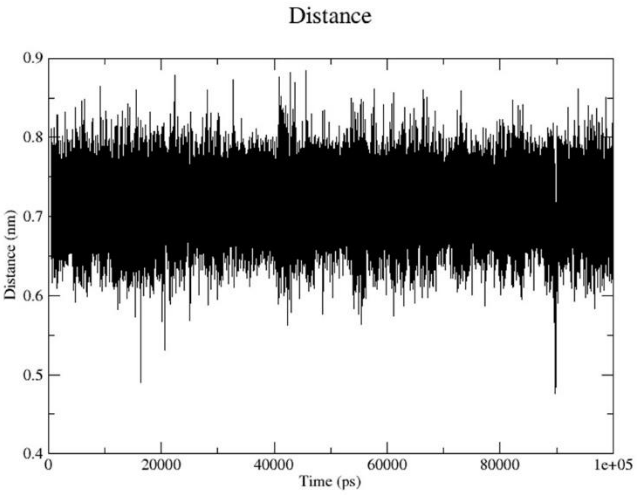


**AZAP-CG1**

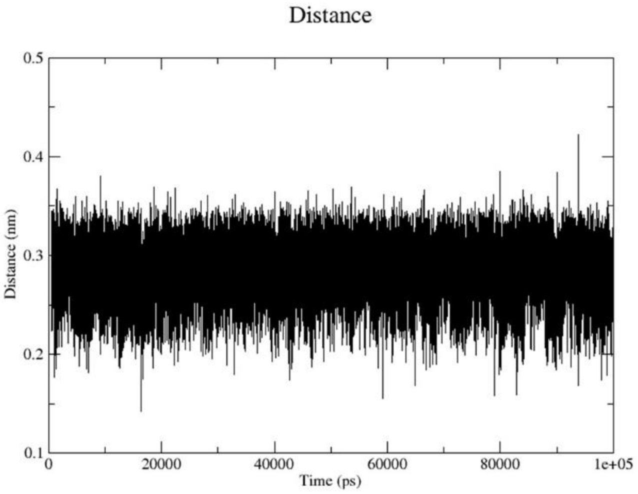


CB[10]-**AZAP**

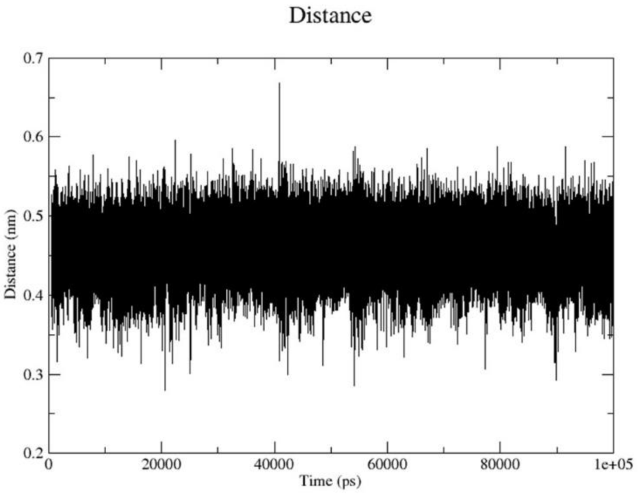


CB[10]-**CG1**

***Figure S26***. Distances between the centre of each component of the complex CB[10]•**AZAP•CG1**.

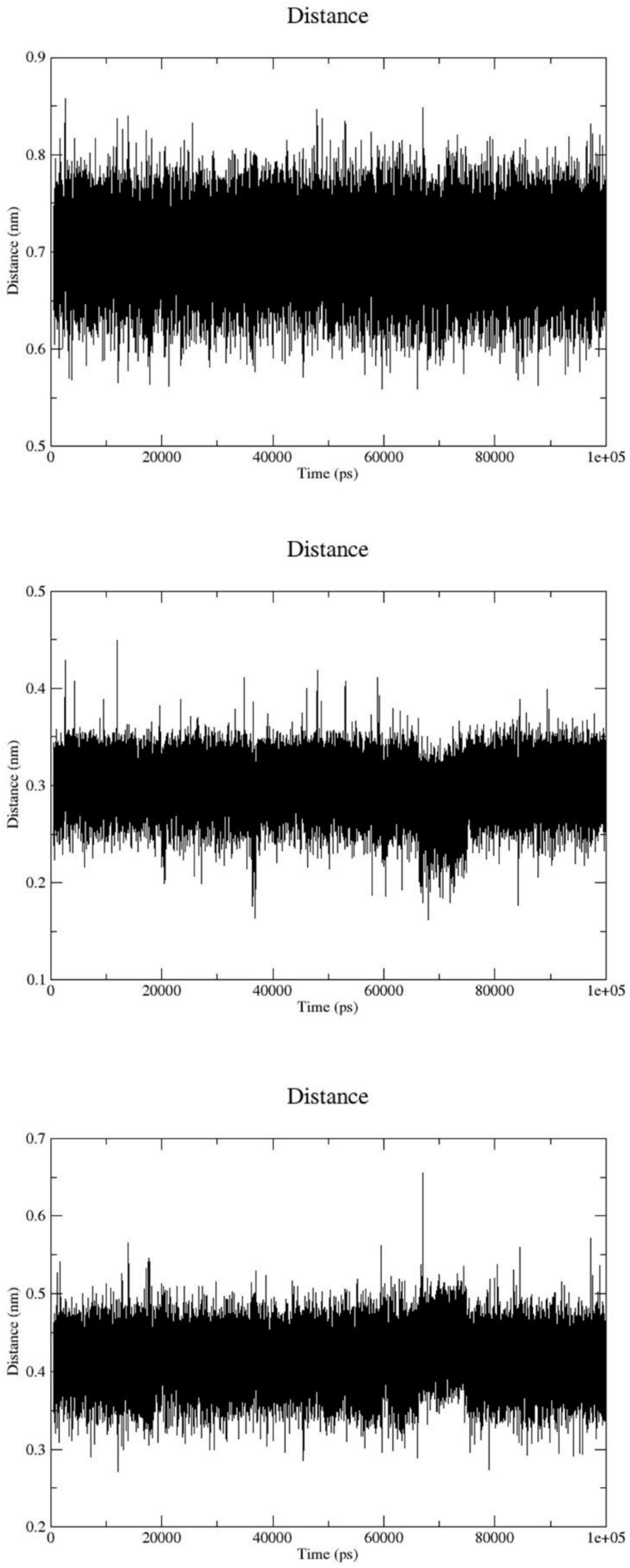


**AZAP-CG2**

CB[10]-**AZAP**

CB[10]-**CG2**

***Figure S27***. Distances between the centre of each component of the complex CB[10]•**AZAP•CG2**.

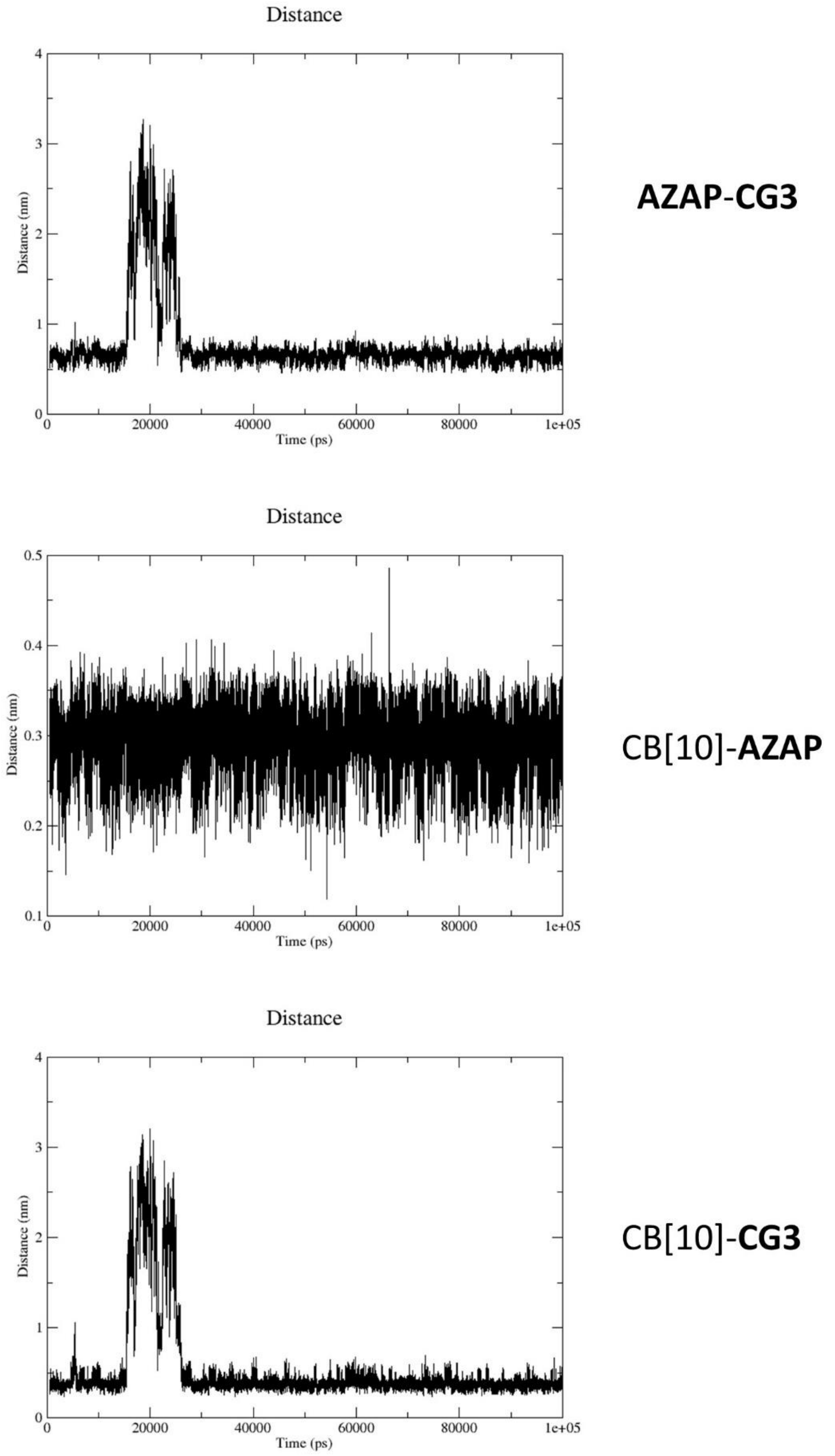


***Figure S28***. Distances between the centre of each component of the complex CB[10]•**AZAP•CG3**.

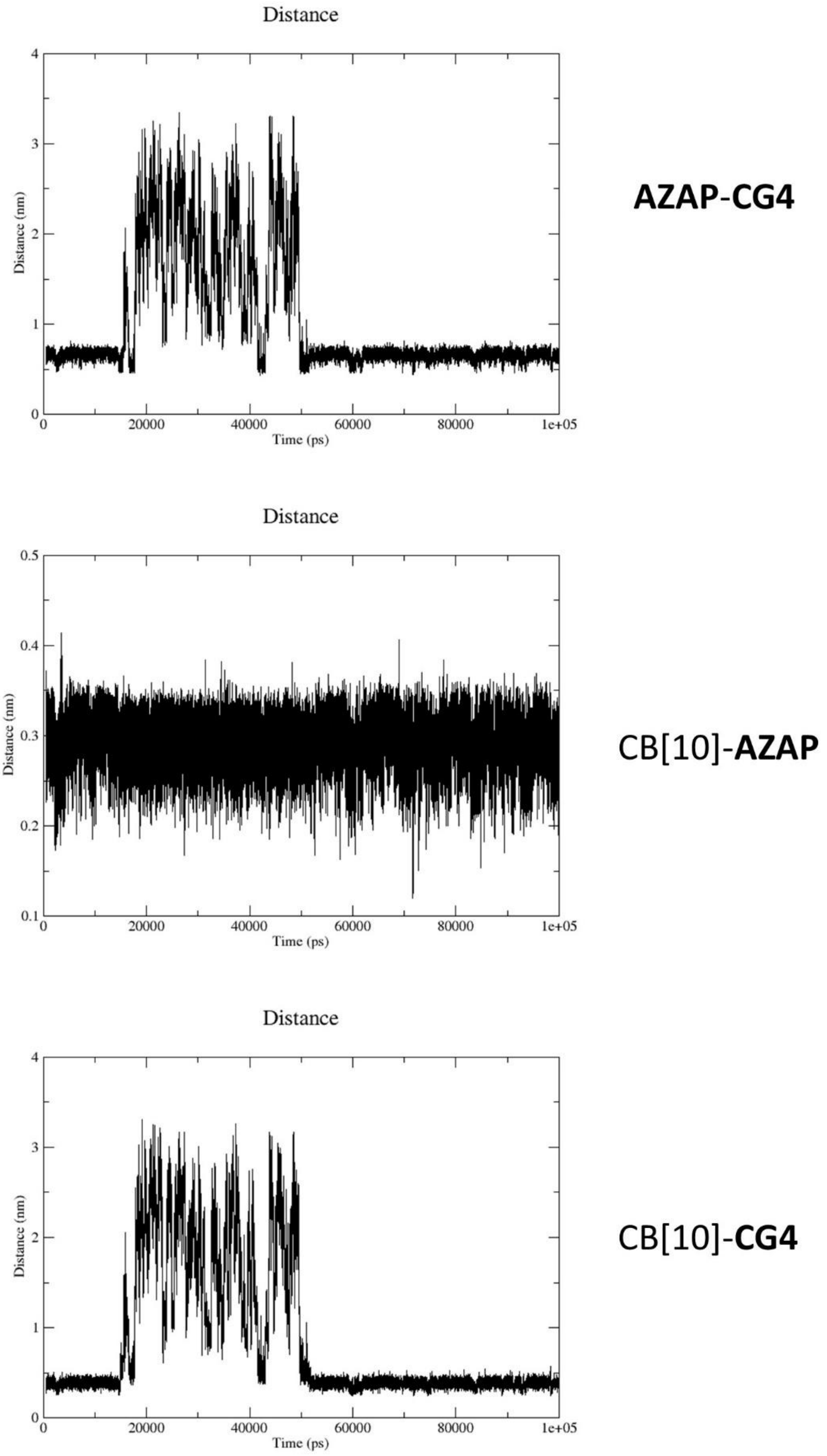


***Figure S29***. Distances between the centre of each component of the complex CB[10]•**AZAP•CG4**.

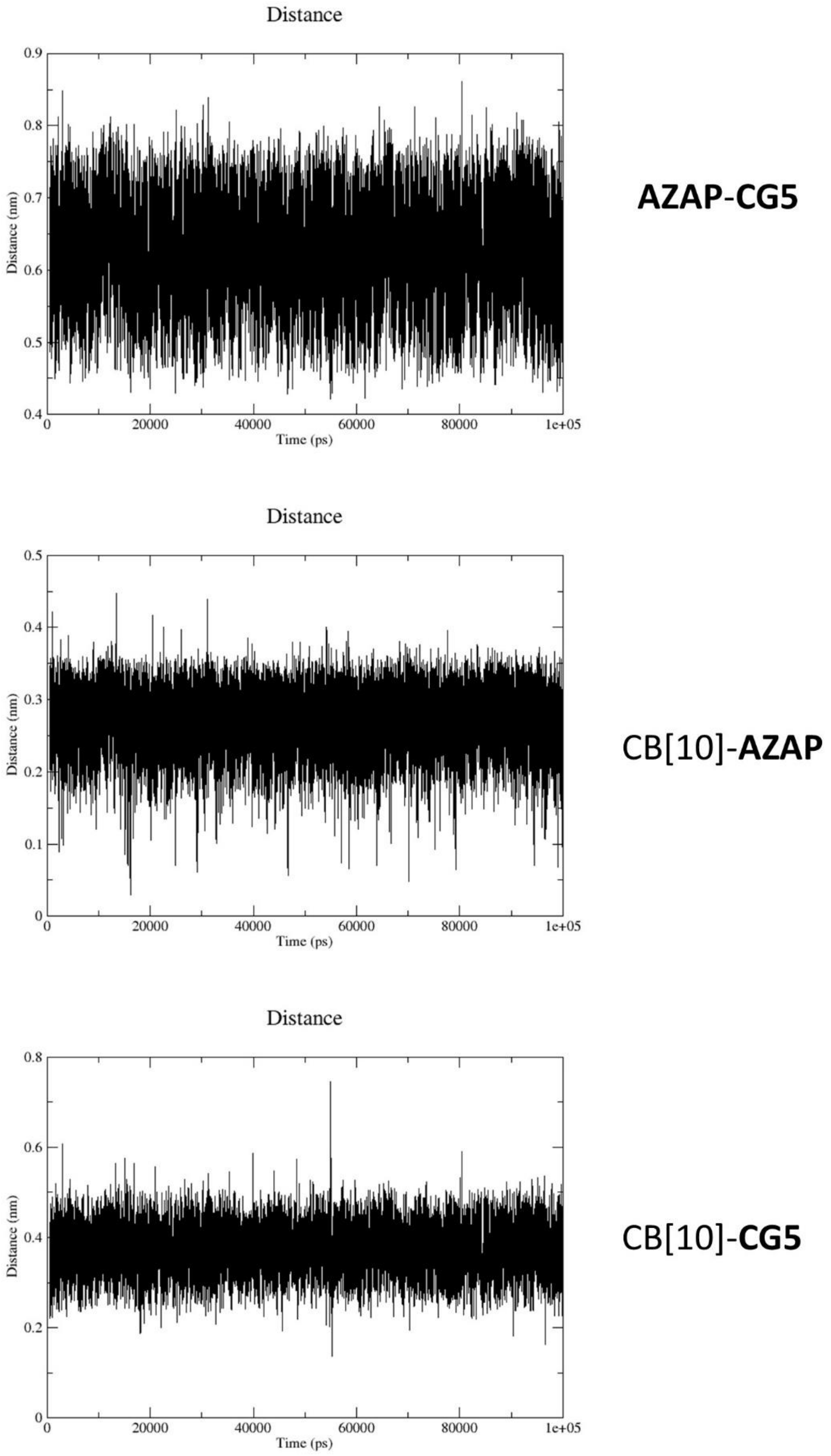


***Figure S30***. Distances between the centre of each component of the complex CB[10]•**AZAP•CG5**.

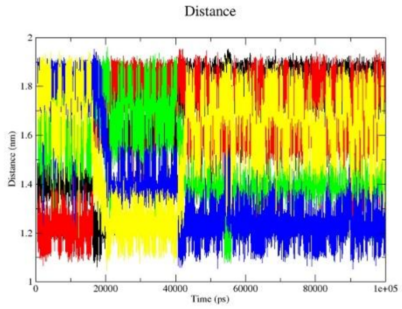


**CG1**

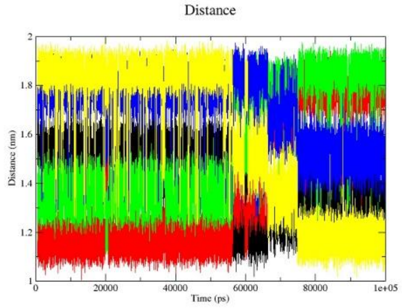


**CG2**

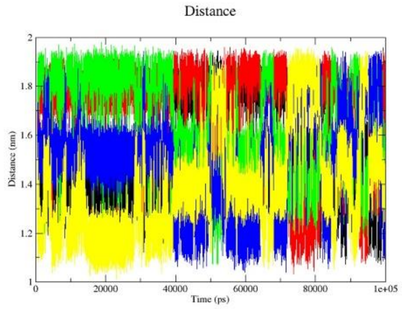


**CG3**

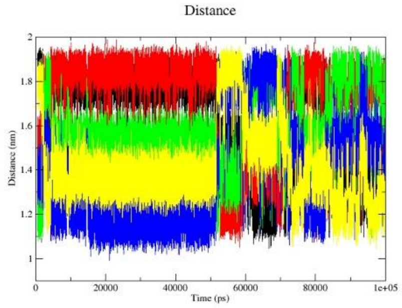


**CG4**

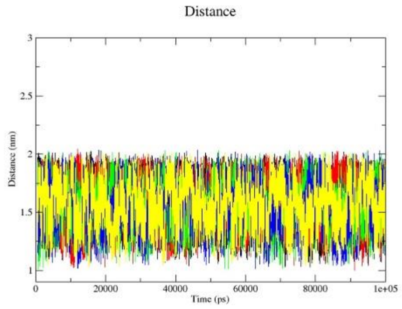


**CG5**

***Figure S31***. Distances between opposite carbon atoms of CB[10] chosen pairwise (in the mean plane orthogonal to the host $C_{10}$ axis) to account for the macrocycle deformation in heteroternary CB[10]•**AZAP•CGx** complexes.

### *32/ References.*


[1] X. Yang, Z. Zhao, X. Zhang and S. Liu, *Sci. China: Chem.* **2018**, *61*, 787-791.

[2] H. Schmidt, C. Lensink, S. K. Xi, J. G. Verkade, *Z. Anorg. Allg. Chem.* **1989**, *578*, 75–80.

[3] M. J. Abraham, T. Murtola, R. Schulz, S. Páll, J. C. Smith, B. Hess, E. Lindahl. *SoftwareX* **2015**, *1*, 19–25.

[4] W. L. Jorgensen, J. D. Madura, *J. Am. Chem. Soc.* **1983**, *105*, 1407–1473.

[5] J. Wang, P. Cieplak, P. A. Kollman, *J. Comput. Chem.* **2000**, *21*, 1049–1074.

[6] M. Mihorianu, M. H. Franz, P. G. Jones, M. Freytag, G. Kelter, H.-H. Fiebig, M. Tamm, I. Neda, *Appl. Organomet. Chem.* **2016**, *30*, 581–589.

[7] A. R. Katritzky, D. Jishkariani, R. Sakhuja, C. D. Hall, P. J. Steel, *J. Org. Chem.* **2011**, *76*, 4082–4087.

[8] J. R. Carreon, K. P. Mahon, S. O. Kelley, *Org. Lett.* **2004**, *4*, 517–519.

[9] M. J. van Haren, J. S. Torano, D. Sartini, M. Emanuelli, R. B. Parsons, N. I. Martin, *Biochemistry* **2016**, *55*, 5307–5315.

[10] X. Yang, Q. Cheng, V. Monnier, L. Charles, H. Karoui, O. Ouari, D. Gigmes, R. Wang, A. Kermagoret, D. Bardelang, *Angew. Chem., Int. Ed.* **2021**, 60, 6617–6623.
